# Discrimination of SARS-CoV-2 omicron variant and its lineages by rapid detection of immune-escape mutations in spike protein RBD using asymmetric PCR-based melting curve analysis

**DOI:** 10.1186/s12985-023-02137-5

**Published:** 2023-08-25

**Authors:** Xiaomu Kong, Peng Gao, Yongwei Jiang, Lixia Lu, Meimei Zhao, Yi Liu, Guoxiong Deng, Haoyan Zhu, Yongtong Cao, Liang Ma

**Affiliations:** Department of Clinical Laboratory, China-Japan Friendship Hospital, No. 2 Yinghua East Street, Chaoyang District, Beijing, 100029 People’s Republic of China

**Keywords:** Omicron, Mutation detection, Asymmetric PCR, Melting curve analysis, SARS-CoV-2

## Abstract

**Background:**

The SARS-CoV-2 Omicron strain has multiple immune-escape mutations in the spike protein receptor-binding domain (RBD). Rapid detection of these mutations to identify Omicron and its lineages is essential for guiding public health strategies and patient treatments. We developed a two-tube, four-color assay employing asymmetric polymerase chain reaction (PCR)-based melting curve analysis to detect Omicron mutations and discriminate the BA.1, BA.2, BA.4/5, and BA.2.75 lineages.

**Methods:**

The presented technique involves combinatory analysis of the detection of six fluorescent probes targeting the immune-escape mutations L452R, N460K, E484A, F486V, Q493R, Q498R, and Y505H within one amplicon in the spike RBD and probes targeting the *ORF1ab* and *N* genes. After protocol optimization, the analytical performance of the technique was evaluated using plasmid templates. Sensitivity was assessed based on the limit of detection (LOD), and reliability was assessed by calculating the intra- and inter-run precision of melting temperatures (T_m_s). Specificity was assessed using pseudotyped lentivirus of common human respiratory pathogens and human genomic DNA. The assay was used to analyze 40 SARS-CoV-2–positive clinical samples (including 36 BA.2 and 4 BA.4/5 samples) and pseudotyped lentiviruses of wild-type and BA.1 viral RNA control materials, as well as 20 SARS-CoV-2–negative clinical samples, and its accuracy was evaluated by comparing the results with those of sequencing.

**Results:**

All genotypes were sensitively identified using the developed method with a LOD of 39.1 copies per reaction. The intra- and inter-run coefficients of variation for the T_m_s were ≤ 0.69% and ≤ 0.84%, with standard deviations ≤ 0.38 °C and ≤ 0.41 °C, respectively. Validation of the assay using known SARS-CoV-2–positive samples demonstrated its ability to correctly identify the targeted mutations and preliminarily characterize the Omicron lineages.

**Conclusion:**

The developed assay can provide accurate, reliable, rapid, simple and low-cost detection of the immune-escape mutations located in the spike RBD to detect the Omicron variant and discriminate its lineages, and its use can be easily generalized in clinical laboratories with a fluorescent PCR platform.

**Supplementary Information:**

The online version contains supplementary material available at 10.1186/s12985-023-02137-5.

## Introduction

The global coronavirus disease 2019 (COVID-19) pandemic was caused by severe acute respiratory syndrome-coronavirus-2 (SARS-CoV-2) virus, and Omicron, the fifth variant of concern (VOC) designated by the World Health Organization (WHO), has been the prevalent strain of SARS-CoV-2 virus in circulation since November, 2021, causing major outbreaks around the world. Recent computational and whole-genome sequencing analyses have identified five main lineages of the Omicron variant descending from an original Omicron ancestor, which are designated BA.1, BA.2, BA.3, BA.4 and BA.5, and several sub-lineages, including BA.1.1, BA.2.12.1, BA.2.11, BA.2.75, BA.4.6 [1–3]. Currently, BA.4/5 has overtaken BA.2 to become the dominant strain and has spread widely in many countries worldwide.

Vaccination has been proven to be the most effective way to prevent and control COVID-19 [4, 5]. The spike protein of the SARS-CoV-2 virus is the main target of antibodies generated by either infection or vaccination. The receptor-binding domain (RBD) of the spike protein is responsible for recognizing and binding to the host angiotensin-converting enzyme 2 (ACE2), which enables entry of the virus into the host human cell followed by initiation of the viral infection process. Thus, the RBD of the spike protein is the key target of vaccines and antibody drugs. Mutations on the spike protein RBD determine the essential infectivity and immune evasion capability of each SARS-CoV-2 variant.

Compared to previous SARS-CoV-2 VOC strains (Alpha, Beta, Gamma, and Delta), Omicron demonstrates enhanced transmissibility and can evade RBD-targeted neutralizing antibodies, due to RBD mutations promoting contagiousness and vaccine escape [6]. Unprecedentedly, more than 32 mutations were identified in the spike protein of Omicron B.1.1.529 (BA.1), including 15 amino-acid substitutions located in the RBD (G339D, S371L, S373P, S375F, K417N, N440K, G446S, S477N, T478K, E484A, Q493R, G496S, Q498R, N501Y, and Y505H). Previous studies have explored the effect of Omicron mutations on viral immune escape through multiple bioinformatics approaches and experimental studies [6–14]. For example, applying an artificial intelligence (AI) model, researchers predicted that Omicron has a high potential to disrupt binding of most antibodies to the spike protein, mainly due to RBD mutations K417N, E484A, and Y505H, indicating a stronger vaccine breakthrough capability than the Delta variant [11]. Moreover, the K417N, E484A, and Q493R mutations reduce the efficacy of the Eli Lilly monoclonal antibody cocktail, and the E484A, Q493R, and Q498R mutations may disrupt the Celltrion antibody drug Regdanvimab [11]. The study applying a combined approach of immunoinformatics and binding free energy calculations evaluated the effect of mutations on the binding affinities of different classes of RBD-specific antibodies. It demonstrated that K417N and Y505H are primarily accountable for the loss of class I antibody binding affinities, and E484A drastically reduces binding affinities for most of the class II antibodies [8]. Another study using molecular dynamics simulations combined with the molecular mechanics-generalized Born surface area method indicated that E484A and Y505H can reduce the binding affinities to RBD for most of the studied neutralizing antibodies [9]. Findings by deep mutational scanning approach supported that fourteen mutations in Omicron may affect antibody binding [10]. Experimental results indicated that mutations at the Y449, E484, Q493, S494, and Y505 sites can enable the virus to escape antibodies [12]. Researchers demonstrated that the neutralizing antibodies are largely escaped via the K417N, G446S, E484A, and Q493R mutations [6]. Moreover, different Omicron lineages carrying novel spike RBD mutations or reverse mutations are constantly emerging and changing the characteristics of the virus. For example, BA.4/5 gained mutations L452R and F486V located in the RBD, which facilitate escape from some antibodies, and a reversion mutation R493Q, which restores viral affinity [15]. The G446S and N460K mutations of BA.2.75 are primarily responsible for its enhanced resistance to neutralizing antibodies, whereas the R493Q mutation reduces its neutralization resistance [16]. Therefore, identification of the Omicron variant and its lineages according to the genotype of key RBD mutations, especially the immune-escape mutations, could be valuable for precise therapeutic management of infected individuals.

At present, identification of SARS-CoV-2 variants and lineages mainly relies on viral whole-genome sequencing (WGS) by next-generation sequencing (NGS) [17]. However, this method is inaccessible in limited-resource settings, and the experimental procedure is labor- and time- consuming. To solve this problem, several techniques based on a polymerase chain reaction (PCR) platform have been developed to rapidly identify spike signature mutations, including high-resolution melting (HRM) analysis [18–20], dual hybridization probe melting analysis [21, 22], and real-time PCR [23–30]. In addition, methods based on Sanger sequencing [31], nanopore-sequencing [32], a CRISPR-based system [33–36], matrix-assisted laser desorption ionization-time of flight mass spectrometry (MALDI-TOF MS) [37], and ion-pair reversed-phase high performance liquid chromatography (IP-RP-HPLC) [38] have been developed for variant detection. The majority of these methods were designed to discriminate SARS-CoV-2 VOC strains, and still few techniques have been developed to discriminate Omicron lineages.

In the present study, we developed a two-tube, four-color assay that uses asymmetric PCR-based melting curve analysis to detect seven immune-escape RBD mutations of Omicron, in order to provide an accurate, stable, rapid, simple, inexpensive, and open-source method for the preliminary identification of the Omicron variant and discrimination of the BA.1, BA.2, BA.4/5, and BA.2.75 lineages, which have been prevalent in the mainland of China. In the future, this technique can be easily applied and generalized as a public health strategy for managing COVID-19. The ability to identify immune-escape RBD mutations also may be useful for guiding precision therapy and predicting prognosis.

## Materials and methods

### Clinical samples

RNA was extracted from the clinical nasopharyngeal swabs using an RNA extraction kit (Tianlong Science and Technology Co., Ltd., Xi’an, China) according to the manufacturer’s instruction. The sample input and output volumes were 200 μL and 80 μL, respectively. RNA extracts were tested for SARS-CoV-2 by quantitative reverse transcription-PCR (RT-PCR). Then, we obtained the remnant RNA extracts which had been tested positive (n = 40) and negative (n = 20) for SARS-CoV-2 for clinical validation of the current methodology. The RNA extracts were stored at –80 °C before use. The study protocol was implemented in accordance with the Declaration of Helsinki II and with approval from the ethics committee of China-Japan Friendship Hospital (Identifier: 2021-149-K107; the date of the approval: Nov 22nd, 2021).

### Plasmid construction

Plasmids with genome fragments of SARS-CoV-2 were obtained from Tsingke Co. Ltd (Beijing, China). Fragments containing the 1129 ~ 1584 or 1212 ~ 1584 coding sequence of the spike genes of the original wild-type virus (Wuhan) and the BA.1, BA.2, BA.4/5, and BA.2.75 lineages of the Omicron variant, as well as the 481 ~ 830 coding sequence in the *N* gene and 12,961–13,310 coding sequence in the *ORF1ab* gene were inserted into the plasmid pUC57, separately. The sequences inserted in the plasmids are presented in Additional file 1: Table S1.

The plasmids were dissolved in TE buffer (10 mmol/L Tris and 0.1 mmol/L EDTA, pH 8.0) to 2 μg/mL. They were further diluted using nuclease-free water (Ambion, Life Technologies Corp., TX, USA) to 40,000 copies/μL. Viral lineages were mimicked by mixing wild-type or mutant spike gene plasmids with *ORF1ab* plasmid and *N* plasmid equally, and diluted to a final concentration of 10,000 copies/μL of each plasmid. The plasmid mixtures were stored at –20 °C.

### Pseudotyped lentivirus

Pseudotyped lentiviruses with SARS-CoV-2 wild-type virus (RNA weakly positive quality control reference material) and Omicron BA.1 virus (quality control reference material) were obtained from Guangzhou BDS Biological Technology Co., Ltd. (Guangzhou, China). Pseudotyped lentiviruses with influenza virus A (Flu A), influenza virus B (Flu B) and respiratory syncytial virus (RSV) were obtained from American Type Culture Collection (VA, USA) for analysis of the analytical specificity of the developed method. Viral RNA was extracted using an RNA extraction kit (Tianlong Science and Technology Co., Ltd.) with an input volume of 200 μL and output volume of 80 μL.

### Reverse transcription of RNA

The extracted RNA was used to synthesize random hexamer-primed cDNA with M-MuLV reverse transcriptase (#K1622, Thermo Fisher Scientific, MA, USA) according to the manufacturer’s instruction. The cDNA was then used for PCR experiments.

### Asymmetric PCR-based melting curve analysis

The developed method is a two-tube, four-color assay that allows simultaneous detection of seven mutations associated with Omicron, permitting discrimination of the wild-type variant, BA.1, BA.2, BA.4/5, and BA.2.75. The *ORF1ab* or *N* gene was detected in each tube as an internal control. Three pairs of primers and eight dual-labeled self-quenched fluorescent probes were designed to detect spike mutations L452R (c.1355 T > G), N460K (c.1380 T > G), E484A (c.1451A > C), F486V (c.1456 T > G), Q493R + Q498R (c.1478A > G + c.1493A > G), and Y505H (c.1513 T > C), along with *ORF1ab* and *N*, respectively (see Table 1). Probes to detect mutations were designed to be completely matched with the mutant sequence of the target mutation sites. The primers and probes were synthesized and purified by Sangon (Shanghai, China).

**Table 1:** List of oligonucleotide primers and probes

Name	5'-Sequence-3'	Size (nt)
Spike gene forward primer	AATCGCTCCAGGGCAAACTG	20
Spike gene reverse primer	AGTTGCTGGTGCATGTAGAA	20
460 K-FAM probe	FAM-AGGAAGTCTAAGCTCAAACCTT-BHQ1	22
486 V-HEX probe	BHQ1-GTGTTAATTGTTACTTTCCT-HEX	20
505H-ROX probe	ROX-TCACCAACCATACAGAGTA-BHQ2	19
484A-FAM probe	FAM-AATGGTGTTGCAGGTTTTA-BHQ1	19
493R + 498R-HEX probe	HEX-CGATCATATGGTTTCCGAC-BHQ1	19
452R-ROX probe	ROX-TACCGGTATAGATTGTTTAGG-BHQ2	21
*ORF1ab* gene forward primer	CCCTGTGGGTTTTACACTTAA*	21
*ORF1ab* gene reverse primer	ACGATTGTGCATCAGCTGA*	19
*ORF1ab*-Cy5 probe	Cy5-TCTGCGGTATGTGGAAAGGTT-BHQ2	21
*N* gene forward primer	GGGGAACTTCTCCTGCTAGAAT*	22
*N* gene reverse primer	CAGACATTTTGCTCTCAAGCTG*	22
*N*-Cy5 probe	Cy5-TTGCTGCTGCTTGACAGATT*-BHQ2	20

*Sequence recommended by China CDC

The method was then optimized and performed using a SLAN®-96P fluorescent quantitative PCR system (Hongshitech, Shanghai, China). The first reaction (reaction 1) contained probes targeting N460K (in FAM channel, BA.2.75 mutation), F486V (in HEX channel, BA.4/5 mutation), and Y505H (in ROX channel, Omicron-specific mutation), with a probe targeting the *ORF1ab* gene (in Cy5 channel) as an internal indicator for SARS-CoV-2 virus. The second reaction (reaction 2) contained probes targeting the spike E484A (in FAM channel, Omicron-specific mutation), Q493R + Q498R (in HEX channel, Omicron-specific mutations, Q493R is not present in BA.4/5 and BA.2.75), and L452R (in ROX channel, BA.4/5 mutation), with a probe targeting the *N* gene (in Cy5 channel) as an internal indicator. Each 25-μL PCR mixture contained TaKaRa Ex Taq® Polymerase (0.125 U/μL) supplied in 10 × Ex Taq Buffer (pH 8.5), dNTP (200 μM each) and 2 mM MgCl_2_ (RR01AM, TaKaRa Bio, Inc., Dalian, China), with 0.1–0.3 μM limiting primers, 0.8–2.4 μM excess primers, 0.1–0.4 μM probes, and 5 μL plasmids or cDNA template. The concentration details for the primers and probes are presented in Additional file 2: Table S2. Moreover, for detection of a single spike mutation, the *ORF1ab* gene, or the *N* gene, 0.1 μM restriction primer, 0.8 μM excess primer, and 0.2 μM probe were used in the reaction.

The PCR cycling program consisted of initial denaturation at 95 °C for 5 min followed by 50 cycles of denaturation at 95 °C for 20 s, annealing at 60 °C for 1 min, and extension at 72°C for 30 s for amplification. The amplified products for spike gene, *ORF1ab* and *N* gene were 343 bp, 119 bp, and 99 bp, respectively. After the amplification procedure, the melting curve program included three steps: denaturation at 95 °C for 2 min, renaturation at 40 °C for 2 min, and subsequent melting with continuous acquisition of fluorescence from 40 to 80 °C at a ramp rate of 0.08 °C/s.

The results were then interpreted via combinatory analysis of the four fluorescence signals of each reaction (see Table 2). In brief, the melting curves for the *ORF1ab* gene (Cy5 channel in reaction 1) and *N* gene (Cy5 channel in reaction 2) suggested the existence of SARS-CoV-2 virus, and the T_m_ of each melting curve in the FAM, HEX, and ROX channels indicated the genotype of the targeted mutations.

**Table 2:** Detection of Omicron spike gene mutations using the asymmetric PCR melting curve analysis-based method

Reaction 1							
Sequence	Probe-460 K-FAM		Probe-486 V-HEX		Probe-505H-ROX		Probe-*Orf1ab*-Cy5
	Genotype	T_m_ Mean ± SD, °C	Genotype	T_m_ Mean ± SD, °C	Genotype	T_m_ Mean ± SD, °C	T_m_ Mean ± SD, °C
Wild type	460N	58.89 ± 0.19	486F	47.82 ± 0.21	505Y	52.95 ± 0.29	64.79 ± 0.32
BA.1	460N	58.93 ± 0.17	486F	47.70 ± 0.16	505H	57.15 ± 0.23	64.79 ± 0.36
BA.2	460N	58.93 ± 0.16	486F	47.72 ± 0.22	505H	57.29 ± 0.22	64.81 ± 0.34
BA.4/5	460N	58.89 ± 0.15	486V	55.47 ± 0.17	505H	57.29 ± 0.23	64.76 ± 0.33
BA.2.75	460 K	63.56 ± 0.18	486F	47.83 ± 0.17	505H	57.34 ± 0.23	64.83 ± 0.36

Reaction 2							
Sequence	Probe-484A-FAM		Probe-493R + 498R-HEX		Probe-452R-ROX		Probe-*N*-Cy5
	Genotype	T_m_ Mean ± SD, °C	Genotype	T_m_ Mean ± SD, °C	Genotype	T_m_ Mean ± SD, °C	T_m_ Mean ± SD, °C
Wild type	484E, 486F	46.58 ± 0.36	493Q, 496G, 498Q	49.34 ± 0.12	452L	51.16 ± 0.15	64.35 ± 0.34
BA.1	484A, 486F	58.42 ± 0.12	493R, 496S, 498R	54.78 ± 0.16	452L	51.21 ± 0.16	64.44 ± 0.17
BA.2	484A, 486F	58.41 ± 0.10	493R, 496G, 498R	61.12 ± 0.13	452L	51.15 ± 0.18	64.37 ± 0.14
BA.4/5	484A, 486V	52.71 ± 0.16	493Q, 496G, 498R	55.50 ± 0.27	452R	57.65 ± 0.15	64.44 ± 0.18
BA.2.75	484A, 486F	58.39 ± 0.14	493Q, 496G, 498R	54.94 ± 0.29	452L	51.18 ± 0.21	64.40 ± 0.15

### Analytical sensitivity

The analytical sensitivity of the presented method was evaluated by examining its performance with varying amounts of plasmid templates at 1250, 625, 312.5, 156.3, 78.1, 39.1, 19.5, and 9.8 copies per reaction via doubling dilution. For each plasmid mixture represented for the variants and lineages, each concentration was analyzed in 20 technical repeats. The limit of detection (LOD) of each reaction was defined as the copy number of the input template that provided the appearance of a melting peak for all targets in > 95% technical repeats.

### Precision

Intra-assay precision was assessed for the plasmid mixtures representing each SARS-CoV-2 virus lineage with an amount of 312.5 copies per reaction in five replicates to determinate the percent coefficient of variance (CV) of T_m_s. Inter-assay precision of T_m_s was assessed based on the results for five replicates of each plasmid mixture tested on four independent days by different operators.

### Analytical specificity

The presented method was tested using RNA extracts of pseudotyped lentiviruses with Flu A, Flu B, and RSV as well as human genomic DNA, to verify that it did not detect either viral nucleic acid from these other widespread human viral pathogens causing respiratory infections or human genomic DNA.

### Accuracy

To assess the accuracy, the presented method was used to test clinical samples for which the status was known, as well as SARS-CoV-2 wild-type RNA weakly positive quality control reference material and Omicron BA.1 quality control reference material. The mutation genotyping results for the positive samples were compared with Sanger sequencing results.

For Sanger sequencing, the primers used to amplify the targeted region of the spike gene in SARS-CoV-2 virus were designed as follows: 5’-AATCGCTCCAGGGCAAACTG-3’ (forward primer), and 5’-AGTTGCTGGTGCATGTAGAA-3’ (reverse primer; Sangon Biotech Co., Ltd.). PCR was carried out in a 50-μL reaction mixture containing TaKaRa Ex Taq® Polymerase (0.125 U) in 10 × Ex Taq Buffer (pH 8.5) with dNTP (200 μM each), 2 mM MgCl_2_ (RR01AM, TaKaRa Bio, Inc.), 0.5 μM forward primer, and 0.5 μM reverse primer. The PCR cycling program consisted of initial denaturation at 95 °C for 5 min followed by 50 cycles of 95 °C for 20 s, 60 °C for 1 min and 72 °C for 30 s, with a final extension at 72 °C for 10 min (C1000 TouchTM Thermal Cycler, Bio-Rad Laboratories, Inc., CA, USA). The amplicons were 343 bp and were sent to Tsingke Biotechnology Co., Ltd. (Beijing, China) for unidirectional sequencing using an ABI 3730xl DNA Analyzer (Applied Biosystems, CA, USA).

### Statistics

The data are presented as mean ± standard deviation (SD), and CVs were calculated using SAS (version 9.3; SAS Institute, NC, USA).

## Results

### Design and establishment of the asymmetric PCR-based melting curve method

**Fig. 1:**
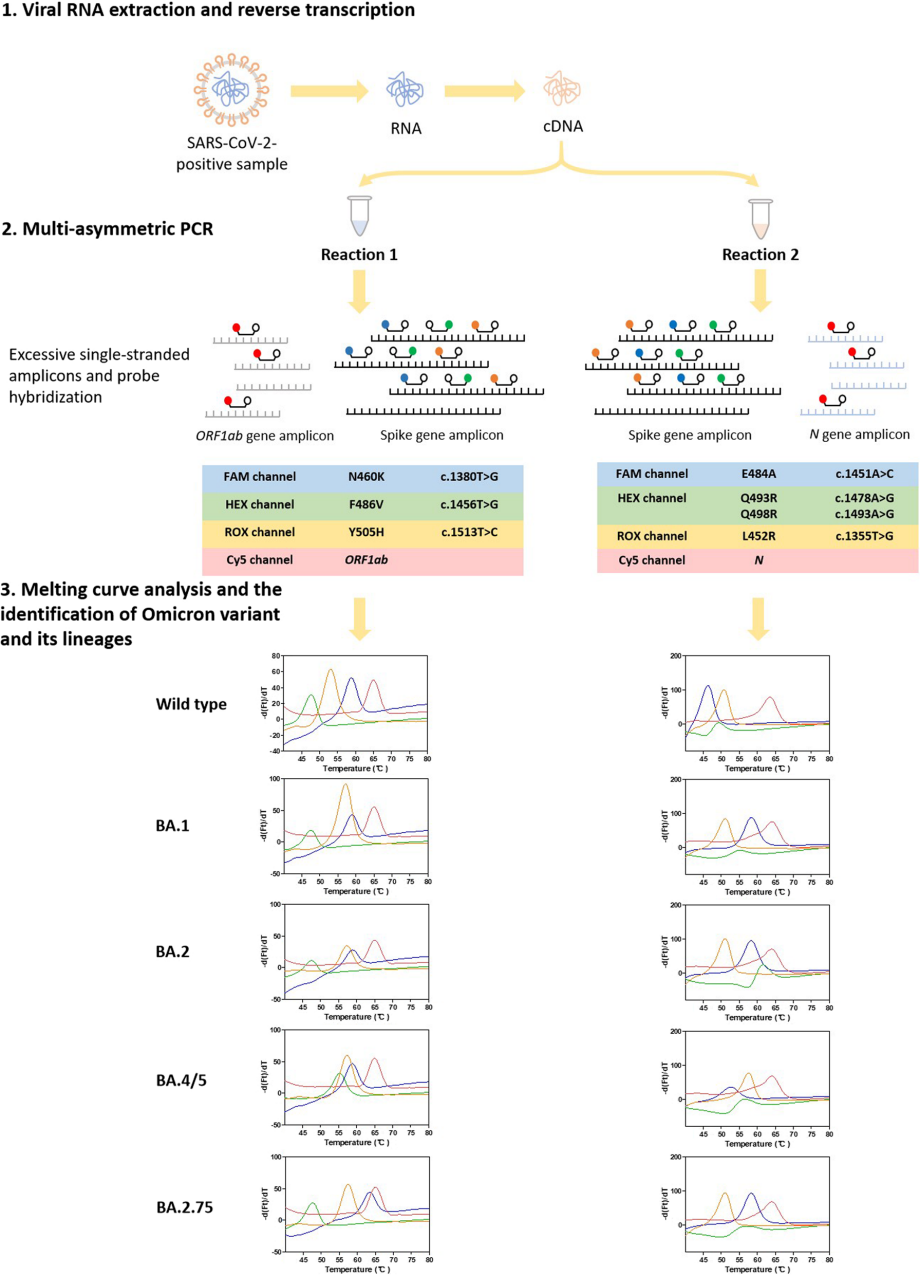
Flow chart of the asymmetric PCR melting curve analysis-based method. SARS-CoV-2 virus RNA is extracted from clinical positive samples. Reverse transcription is performed to generate cDNA from extracted virus RNA. Excessive single-stranded amplicons are obtained from multi-asymmetric PCR. In the first tube (reaction 1), spike gene amplicon and *ORF1ab* gene amplicon are generated, and probes targeting spike RBD mutation N460K (FAM), F486V (HEX), Y505H (ROX) and the *ORF1ab* gene (Cy5) are hybridized to the single-stranded products. In the second tube (reaction 2), spike gene amplicon and *N* gene amplicon are generated, and probes targeting E484A (FAM), Q493R + Q498R (HEX), L452R (ROX) and the *N* gene (Cy5) are hybridized to the amplicons. The representative graphic outputs of reaction 1 and reaction 2 for the wild-type, Omicron BA.1, BA.2, BA.4/5, and BA.2.75 sequences are shown in the lower panel. The melting curve plots for spike RBD mutation sites 460, 486, 505 and the *ORF1ab* gene are indicated in blue, green, orange and red lines respectively for reaction 1. The melting curve plots for spike RBD mutation sites 484, 493 + 498, 452 and the *N* gene are indicated in blue, green, orange and red lines for reaction 2. Then, the genotypes and final identification of Omicron variant and its lineages can be interpreted by combinatory analysis of the T_m_s of the four melting curves in each reaction (see Table 2)

Figure 1 presents the workflow of the developed method in clinical test. In brief, viral RNA is extracted, and then cDNA is generated after reverse transcription and added to PCR tubes. Then, asymmetric PCR and melting curve analysis are performed as programmed in two tubes with four colors. Asymmetric PCR is used to obtain excess copies of single-stranded amplicons, and probes with different fluorescence tags are hybridized to the targeted amplicons at low temperature and later dissociated as the temperature increases during the melting analysis process. The sequences completely matched with the probe dissociate at higher temperature, whereas the sequences not completely matched dissociate with the probe at lower temperature. When completed, the graphic outputs with T_m_ values are automatically generated.

The experimental results presented in Table 2 demonstrate that the method can accurately detect seven spike RBD mutations and easily discriminate the wild-type virus and the Omicron BA.1, BA.2, BA.4/5, and BA.2.75 lineages from each other. Meanwhile, all seven mutations were covered in the same 343-bp amplicon, which can be generated from an identical primer pair. We first tested each probe individually (see Additional file 3: Table S3, Additional file 4: Fig. S1) and then observed good performance after combining the probes in two tubes. After protocol optimization, reaction 1 detects N460K, F486V and Y505H with the *ORF1ab* gene as the internal control, while reaction 2 detects E484A, Q493R + Q498R and L452R with the *N* gene as the internal control. As expected, T_m_s of probes in different combinations were usually slightly changed compared with those in single tests (see Table 2, Additional file 3: Table S3).

As shown in Table 2, the mean T_m_s for the *ORF1ab* peak ranged from 64.76 to 64.83 °C for all tested sequences in reaction 1, and the mean T_m_s for the *N* peak were between 64.35 °C and 64.44 °C in reaction 2. These peaks demonstrate the existence of SARS-CoV-2 virus.

**Fig. 2:**
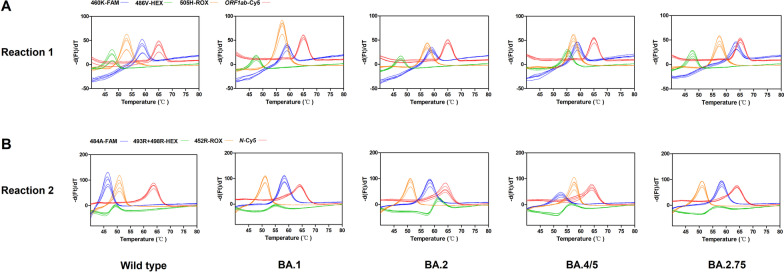
Identification of Omicron variant and its lineages by using the asymmetric PCR melting curve analysis-based method. The performance of reaction 1 (**A**) and reaction 2 (**B**) for wild-type, Omicron BA.1, BA.2, BA.4/5, and BA2.75 with 312.5 template copies per reaction (n = 5). **A** In reaction 1, four colored lines of each sample indicate the melting curves of RBD mutation site 460 (blue), 486 (green), 505 (orange) and the *ORF1ab* gene (red). **B** In reaction 2, the colored lines indicate the melting curves of RBD mutation site 484 (blue), 493 + 498 (green), 452 (orange) and the *N* gene (red)

The genotypes of mutations could be further identified by their corresponding T_m_ values, labeled fluorophores, and reaction number (see Table 2). First, E484A, Q493R, Q498R, and Y505H are Omicron-specific mutations. For wild-type virus, 484E resulted in a mean T_m_ of 46.58 °C, which was clearly distinguishable from the mean T_m_s of the completely matched sequence 484A in BA.1, BA.2, and BA.2.75 (58.39–58.42 °C). Since an additional mutation F486V in BA.4/5 is covered by the probe targeting E484A, its T_m_s was shifted to 52.71 °C. The probe targeting Q493R + Q498R was designed to be completely matched with the haplotype of BA.2 (493R, 496G, 498R) with a T_m_ of 61.12 °C. The mean T_m_ for wild-type virus was significantly decreased to 49.34 °C because of the 2-bp mismatches (493Q, 496G, 498Q). The T_m_ for BA.1 (493R, 496S, 498R) was lower (54.78 °C) than that for BA.2 because of the 1-bp mismatch. The mean T_m_s for BA.4/5 and BA.2.75 were shifted to 55.50 °C and 54.94 °C, respectively, because of the existence of the R493Q reversion mutation. The T_m_s for the BA.1, BA.4/5, and BA.2.75 did not differ significantly, but the respective melting curves had slightly different shapes (see Fig. 2). For the probe targeting Y505H mutation, the mean T_m_s for the Omicron lineages ranged from 57.15 to 57.34 °C, which could be easily distinguished from that for the wild-type virus (52.95 °C).

The BA.4 and BA.5 lineages have identical spike mutations. Relative to BA.2, BA.4/5 have additional RBD mutations, L452R and F486V. The probes targeting L452R and F486V showed significantly higher T_m_s (57.65 °C and 55.47 °C, respectively) for BA.4/5 than for wild-type virus, Omicron BA.1, BA.2, and BA.2.75 (51.15–51.21 °C, 47.70–47.83 °C).

The BA.2.75 lineage has an additional RBD mutation N460K. The probe matching N460K showed a mean T_m_ of 63.56 °C, which was obviously higher than the T_m_s for wild-type virus, Omicron BA.1, BA.2, and BA.4/5 (58.89–58.93 °C).

### Performance

**Fig. 3:**
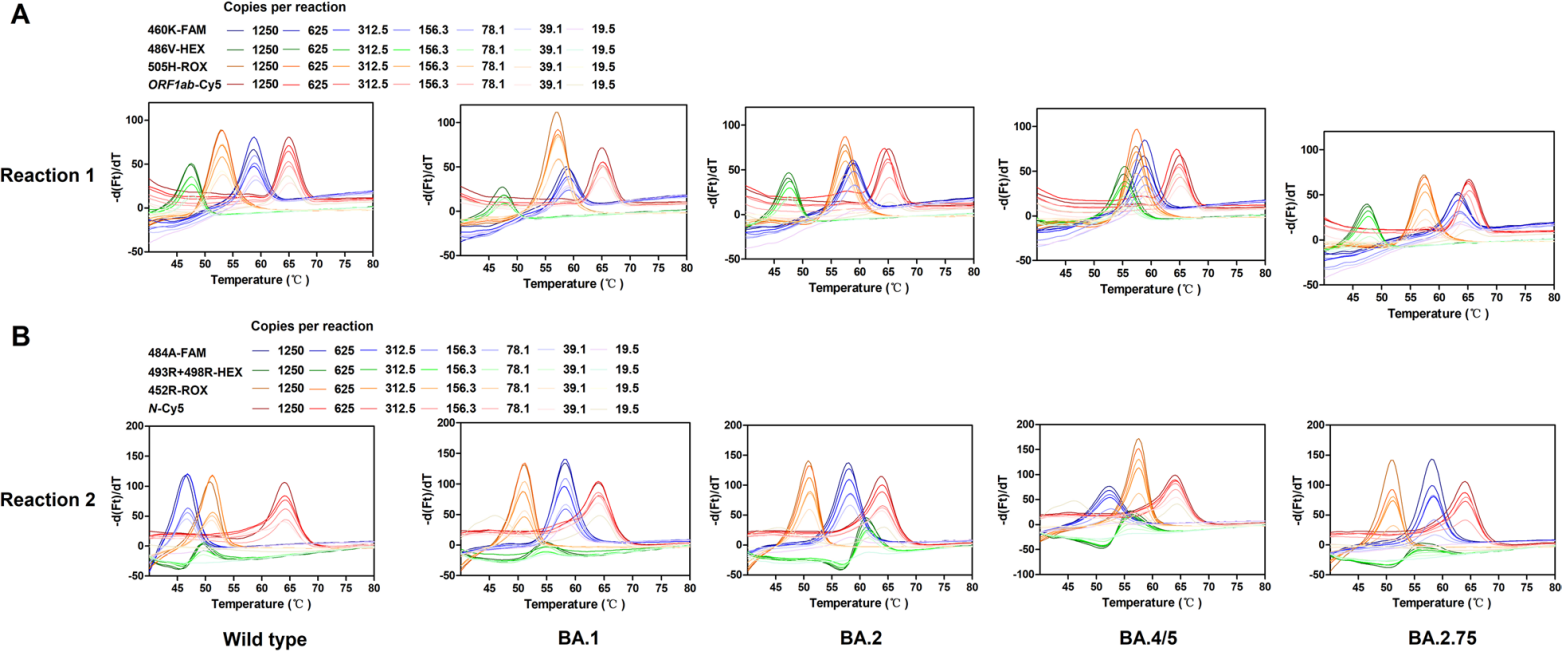
Limit of detection of the asymmetric PCR melting curve analysis-based method. The performance of reaction 1 (**A**) and reaction 2 (**B**) with varying amounts of plasmid templates at 1250, 625, 312.5, 156.3, 78.1, 39.1, and 19.5 copies per reaction are presented. For both reactions, the limit of detect is 39.1 copies per reaction

We use plasmid DNA as the amplification template with varying amounts at 1250, 625, 312.5, 156.3, 78.1, 39.1, 19.5, and 9.8 copies per reaction to assess the analytical sensitivity. The LOD of the present method reached 39.1 copies per reaction for all lineage types, whereas the LODs for each probe in a single test were even lower (see Fig. 3, Additional file 5: Fig. S2).

Five replicates of each genotype were tested on four independent days by different operators to determine the intra-run and inter-run precision. As shown in Table 3, we obtained an intra-run T_m_ CV ≤ 0.69% (range, 0.03%–0.69%) with a SD ≤ 0.38 °C (range, 0.02–0.38 °C) and an inter-run T_m_ CV ≤ 0.84% (range, 0.16%–0.84%) with a SD ≤ 0.41 °C (range, 0.09–0.41°C). The T_m_ values had three times standard deviation (3SD) values ≤ 1.17 °C. All the other genotypes could be successfully resolved with a T_m_ difference larger than 4 °C, which is much larger than the 3SD values, except that the haplotype in BA.1 (493R, 496S, 498R) versus the haplotype of BA.4/5 and BA.2.75 (493Q, 496G, 498R) could not be distinguished by the 493R + 498R probe.

**Table 3:** Inter- and intra-run mean ± SDs precision values of T_m_s

Reaction 1																	
Sequence	Day	Probe-460 K-FAM				Probe-486 V-HEX				Probe-505H-ROX				Probe-*Orf1ab*-Cy5			
		Intra-run T_m_		Inter-run T_m_		Intra-run T_m_		Inter-run T_m_		Intra-run T_m_		Inter-run T_m_		Intra-run T_m_		Inter-run T_m_	
		Mean ± SD, °C	CV (%)	Mean ± SD, °C	CV (%)	Mean ± SD, °C	CV (%)	Mean ± SD, °C	CV (%)	Mean ± SD, °C	CV (%)	Mean ± SD, °C	CV (%)	Mean ± SD, °C	CV (%)	Mean ± SD, °C	CV (%)
Wild type	1	59.19 ± 0.05	0.08	58.89 ± 0.21	0.35	48.13 ± 0.03	0.06	47.82 ± 0.22	0.47	53.32 ± 0.05	0.09	52.95 ± 0.32	0.61	65.16 ± 0.05	0.08	64.79 ± 0.36	0.55
	2	58.73 ± 0.07	0.13			47.65 ± 0.07	0.14			52.67 ± 0.04	0.08			64.48 ± 0.06	0.10		
	3	58.84 ± 0.04	0.06			47.82 ± 0.06	0.13			53.12 ± 0.05	0.10			65.03 ± 0.03	0.05		
	4	58.78 ± 0.08	0.13			47.67 ± 0.07	0.16			52.69 ± 0.04	0.07			64.49 ± 0.06	0.10		
BA.1	1	59.18 ± 0.06	0.11	58.93 ± 0.18	0.30	47.91 ± 0.02	0.04	47.70 ± 0.15	0.31	57.41 ± 0.02	0.03	57.15 ± 0.25	0.44	65.19 ± 0.04	0.05	64.79 ± 0.39	0.61
	2	58.84 ± 0.06	0.10			47.59 ± 0.11	0.23			56.93 ± 0.03	0.06			64.43 ± 0.14	0.21		
	3	58.88 ± 0.07	0.12			47.70 ± 0.08	0.17			57.32 ± 0.05	0.09			65.05 ± 0.08	0.12		
	4	58.79 ± 0.10	0.17			47.59 ± 0.11	0.24			56.93 ± 0.03	0.05			64.47 ± 0.09	0.14		
BA.2	1	59.19 ± 0.09	0.15	58.93 ± 0.17	0.29	47.99 ± 0.06	0.13	47.72 ± 0.20	0.42	57.56 ± 0.06	0.11	57.29 ± 0.24	0.42	65.23 ± 0.04	0.07	64.81 ± 0.38	0.59
	2	58.85 ± 0.07	0.12			47.53 ± 0.22	0.47			57.11 ± 0.05	0.08			64.51 ± 0.06	0.10		
	3	58.88 ± 0.05	0.09			47.72 ± 0.10	0.20			57.43 ± 0.06	0.10			65.03 ± 0.05	0.08		
	4	58.81 ± 0.05	0.09			47.61 ± 0.12	0.25			57.07 ± 0.06	0.11			64.46 ± 0.09	0.14		
BA.4/5	1	59.12 ± 0.06	0.11	58.89 ± 0.16	0.27	55.71 ± 0.08	0.14	55.47 ± 0.18	0.32	57.57 ± 0.03	0.06	57.29 ± 0.26	0.45	65.10 ± 0.16	0.24	64.76 ± 0.35	0.55
	2	58.79 ± 0.06	0.10			55.34 ± 0.06	0.11			57.07 ± 0.03	0.06			64.46 ± 0.08	0.12		
	3	58.85 ± 0.05	0.08			55.49 ± 0.09	0.16			57.44 ± 0.06	0.10			65.03 ± 0.06	0.10		
	4	58.79 ± 0.06	0.10			55.34 ± 0.06	0.11			57.07 ± 0.03	0.06			64.46 ± 0.08	0.12		
BA.2.75	1	63.85 ± 0.05	0.08	63.56 ± 0.20	0.31	48.08 ± 0.12	0.24	47.83 ± 0.17	0.36	57.62 ± 0.04	0.06	57.34 ± 0.26	0.45	65.25 ± 0.04	0.06	64.83 ± 0.41	0.63
	2	63.44 ± 0.03	0.04			47.71 ± 0.05	0.11			57.12 ± 0.05	0.09			64.48 ± 0.06	0.09		
	3	63.53 ± 0.07	0.11			47.83 ± 0.09	0.19			57.50 ± 0.05	0.09			65.11 ± 0.05	0.07		
	4	63.44 ± 0.03	0.04			47.71 ± 0.05	0.11			57.12 ± 0.05	0.09			64.48 ± 0.06	0.09		

Reaction 2																	
Sequence	Day	Probe-484A-FAM				Probe-493R + 498R-HEX				Probe-452R-ROX				Probe-*N*-Cy5			
		Intra-run T_m_		Inter-run T_m_		Intra-run T_m_		Inter-run T_m_		Intra-run T_m_		Inter-run T_m_		Intra-run T_m_		Inter-run T_m_	
		Mean ± SD, °C	CV (%)	Mean ± SD, °C	CV (%)	Mean ± SD, °C	CV (%)	Mean ± SD, °C	CV (%)	Mean ± SD, °C	CV (%)	Mean ± SD, °C	CV (%)	Mean ± SD, °C	CV (%)	Mean ± SD, °C	CV (%)
Wild type	1	46.02 ± 0.06	0.14	46.58 ± 0.39	0.84	49.24 ± 0.03	0.06	49.34 ± 0.12	0.24	51.01 ± 0.03	0.07	51.16 ± 0.15	0.30	64.27 ± 0.18	0.28	64.35 ± 0.36	0.55
	2	46.88 ± 0.04	0.08			49.48 ± 0.07	0.15			51.33 ± 0.05	0.10			64.61 ± 0.05	0.08		
	3	46.82 ± 0.13	0.28			49.40 ± 0.07	0.14			51.24 ± 0.13	0.26			64.64 ± 0.16	0.25		
	4	46.59 ± 0.07	0.15			49.24 ± 0.05	0.09			51.05 ± 0.07	0.14			63.88 ± 0.06	0.09		
BA.1	1	58.29 ± 0.05	0.08	58.42 ± 0.11	0.19	54.79 ± 0.21	0.38	54.78 ± 0.10	0.19	50.97 ± 0.02	0.04	51.21 ± 0.17	0.33	64.25 ± 0.04	0.06	64.44 ± 0.16	0.25
	2	58.44 ± 0.04	0.06			54.64 ± 0.15	0.27			51.25 ± 0.06	0.11			64.57 ± 0.15	0.23		
	3	58.56 ± 0.10	0.18			54.88 ± 0.12	0.23			51.33 ± 0.11	0.21			64.57 ± 0.05	0.08		
	4	58.40 ± 0.06	0.10			54.80 ± 0.04	0.08			51.31 ± 0.06	0.11			64.37 ± 0.12	0.19		
BA.2	1	58.29 ± 0.08	0.14	58.41 ± 0.09	0.16	61.00 ± 0.11	0.18	61.12 ± 0.11	0.18	50.85 ± 0.04	0.08	51.15 ± 0.20	0.39	64.23 ± 0.08	0.12	64.37 ± 0.11	0.17
	2	58.49 ± 0.02	0.03			61.20 ± 0.07	0.12			51.27 ± 0.06	0.11			64.44 ± 0.14	0.21		
	3	58.47 ± 0.08	0.13			61.04 ± 0.11	0.17			51.25 ± 0.08	0.15			64.46 ± 0.12	0.18		
	4	58.41 ± 0.05	0.09			61.22 ± 0.04	0.07			51.22 ± 0.05	0.10			64.33 ± 0.09	0.14		
BA.4/5	1	52.53 ± 0.08	0.14	52.71 ± 0.12	0.24	55.12 ± 0.08	0.15	55.50 ± 0.26	0.46	57.43 ± 0.05	0.09	57.65 ± 0.15	0.26	64.26 ± 0.12	0.18	64.44 ± 0.16	0.25
	2	52.71 ± 0.15	0.28			55.59 ± 0.21	0.38			57.70 ± 0.09	0.15			64.50 ± 0.03	0.05		
	3	52.78 ± 0.18	0.34			55.58 ± 0.20	0.37			57.75 ± 0.06	0.10			64.64 ± 0.16	0.25		
	4	52.81 ± 0.06	0.11			55.69 ± 0.11	0.20			57.73 ± 0.04	0.06			64.36 ± 0.07	0.11		
BA.2.75	1	58.19 ± 0.09	0.15	58.39 ± 0.14	0.24	54.70 ± 0.25	0.46	54.94 ± 0.19	0.35	50.84 ± 0.04	0.09	51.18 ± 0.23	0.44	64.30 ± 0.10	0.15	64.40 ± 0.15	0.23
	2	58.46 ± 0.08	0.14			54.88 ± 0.38	0.69			51.27 ± 0.02	0.04			64.59 ± 0.04	0.06		
	3	58.51 ± 0.08	0.13			55.02 ± 0.18	0.33			51.32 ± 0.09	0.17			64.42 ± 0.13	0.21		
	4	58.40 ± 0.05	0.09			55.15 ± 0.08	0.15			51.27 ± 0.07	0.14			64.27 ± 0.07	0.11		

To assess the specificity of this method, cDNA generated from RNA extracts of pseudotyped lentiviruses of common human respiratory pathogens, including Flu A, Flu B, and RSV, were tested. No cross-reactivity was seen for any of these pathogens in this assay. Moreover, no signal was detected after addition of human genomic DNA.

### Clinical evaluation

The new assay was used to for blinded evaluation of SARS-CoV-2–positive (n = 40) and –negative (n = 20) nasopharyngeal swabs, as well as weakly positive quality control RNA reference material of SARS-CoV-2 wild-type virus and a quality control reference material of Omicron BA.1. No signal was detected from cDNA sample generated from SARS-CoV-2–negative swabs. The results for positive samples were compared with Sanger sequencing results. The developed method detected the *ORF1ab* and *N* gene peaks in the Cy5 channel of all SARS-CoV-2–positive samples with T_m_s ranging from 63.59 to 65.01 °C and from 63.75 to 64.40 °C, respectively. Furthermore, our method accurately identified the wild-type quality control reference material and BA.1 quality control reference material. The current method also correctly classifies all of the BA.2 (n = 36) and BA.4/5 (n = 4) among the 40 positive clinical samples. Complete concordance between the melting curve method and Sanger sequencing results was obtained, demonstrating that the developed method is able to preliminarily distinguish Omicron variants and its lineages (see Additional file 6: Fig. S3, Additional file 7: Table S4, Additional file 8: Table S5).

## Discussion

Since its discovery in December of 2019, the SARS-CoV-2 virus has continuously gained evolutionary advantages in the RBD of its spike protein either by mutations to strengthen the ACE2–RBD binding affinity or by mutations to escape antibody protection, due to natural selection [39]. For the latest VOC strain, Omicron, multiple RBD mutations have accumulatively enhanced its infectivity, suggesting the virus is continuing to optimize its infectivity in human cells. The unprecedent large number of spike mutations suggests that Omicron may have been induced by vaccination, which may dramatically enhance its evasion from current vaccines [11]. Epidemiological studies revealed that the Rt of BA.2 is higher than that of BA.1, while the effective reproduction number (Rt) of BA.4 and BA.5 are higher than that of BA.2. The neutralizing activities against BA.4 and BA.5 by monoclonal antibodies, vaccination and previous SARS-CoV-2 infection were lower than those against BA.1 and BA.2, indicating that the Omicron variant has continued to evolve with increasing immune escape [3]. In the future, it is highly possible that vaccination and neutralizing antibodies will become the main evolutionary pressures on the SARS-CoV-2 virus, and more immune-escape mutations will emerge, especially in the spike RBD region. Any mutation in the RBD region should induce immediate concern about how much the new mutation can undermine the efficacy of existing vaccines and monoclonal antibodies, as well as about the potential for reinfection [11]. Therefore, continuous surveillance of RBD mutations is of great importance. Rapid identification of the immune-escape mutations located in the spike RBD can preliminarily distinguish viral lineages and predict the viral immune evasion ability, which is of clinical significance for guiding both public health interventions and precise immunotherapy regimens for patient infected with a specific strain.

In the present study, we developed an asymmetric PCR-based melting curve assay to rapidly identify the key immune-escape mutations in SARS-CoV-2 and demonstrated that this assay can successfully identify wild-type virus, Omicron BA.1, BA.2, BA.4/5, and BA.2.75 that were once prevalent in the mainland of China. We selected spike RBD mutations in the receptor-binding motif (amino acids 438–506). In this motif, six identical mutations (S447N, T478K, E484A, Q498R, N501Y, and Y505H) are present in all five Omicron main lineages. Among them, E484A, Q498R and Y505H are Omicron-specific mutations. Furthermore, both BA.1 and BA.2 have Q493R, whereas BA.1 has an additional G496S mutation compared to BA.2. BA.4/5 also have L452R, F486V, and a reversion mutation R493Q compared with BA.2 in this motif [3]. BA.2.75, which was increasing in prevalence in southeast Asia and around the world and was most recently prevalent in Hainan Province, China, has an additional mutation N460K and a reversion mutation R493Q compared with BA.2 [16]. Notably, in silico and/or experimental results have revealed that these mutations favor escape from current vaccines or antibodies [6–16, 40]. Thus, these seven signature mutations with immune escape function, including E484A, Y505H, Q493R, Q498R, L452R, F486V, and N460K, were finally selected to develop our novel technique.

Currently, WGS of viruses are routinely used to identify SARS-CoV-2 variants and lineages. However, this is a comprehensive approach for variant detection. The sequencing platform, which requires routine, time-consuming and labor-intensive maintenance, is not available in most clinical laboratories outside of genomic centers. Data interpretation is also challenging and requires experienced technicians. As an alternative to NGS or Sanger sequencing, several groups have attempted to develop PCR-based preliminary methods for variant determination via detection of specific mutations [18–29]. PCR-based methods are rapid, performed simply and applicable, but can only detect the known mutations. Till now, most of the reported RT-PCR-based methods are suitable for the detection of a single mutation, such as the most frequently reported high-resolution melting curve-based analysis or the dual hybridization probe melting analysis [18–22]. There’s still an urgent need to develop the rapid, accurate, economic and accessible method for multiplex sites detection with high-throughput potential, especially for the discrimination of Omicron lineages and immune-escape mutations. Methods based on CRISPR-system, MALDI-TOF MS or IP-RP-HPLC also showed high-throughput potential to detect multiple mutations [33–38]. However, despite being promising approaches, these techniques require certain instrument platforms and have high requirements for the laboratory and the operators, which might be not applicable in many areas at present. Several multiplex quantitative RT-PCR assays were previously reported [23, 24, 26, 29]. Jessen R. and colleagues used the degenerate probes to identify certain mutations, which required two probes for each mutation site to detect the wild type and mutant genotype, respectively. The LOD of the multiplex PCR was around 50 copies/μL of RNA [26]. Xiong D. and colleagues combined the amplification refractory mutation system (ARMS) primers with TaqMan probes, and achieved an LOD as low as 1 copy/μL of RNA [29]. Chung HY. and colleagues employed the double-quenched probes in a multiplex PCR, which positions an internal quencher 9 bases from the 5’ fluorophore and can significantly increases the analytical sensitivity when combined with the 3’ quencher, and achieved LODs between 30–60 copies/μL of purified DNA fragments [24]. Chaintoutis S. and colleagues employed non-extendable locked nucleic acid (LNA) oligonucleotides to reduce non-specific hybridization in the real time RT-PCR-based methodology, and the assay was sensitive to RNA extracts with a LOD of 263 copies per reaction for Delta variant, and 170 copies per reaction for Beta variants [23].

The presented method developed by our group is a two-tube, four-color assay based on a fluorescent PCR platform. This assay requires simple and labor-saving operation and can be easily performed locally in most clinical laboratories. It is basically a reverse transcription and multiple asymmetric PCR analysis. All mutations recognized by 484A, 505H, 486V, 452R, and 460K probes can be successfully resolved with a T_m_ difference larger than 4 °C. Although the haplotype of BA.1 (493R, 496S, 498R) versus the haplotype of BA.4/5 and BA.2.75 (493Q, 496G, 498R) cannot be distinguished from each other by the T_m_s of the 493R + 498R probe in this assay because both genotypes have a 1-bp mismatch compared with the probe, these lineages can be successfully identified based on the results for the other mutations or by applying single probe tests (mean T_m_s of 493R + 498R probe: 53.57 °C for BA.1 and 55.47 °C and 55.35 °C for BA.4/5 and BA.2.75). Thus, the results can be easily determined by both experienced and inexperienced technicians. The clinical validation data further demonstrated that the developed method can accurately genotyped wild-type, BA.1, BA.2, BA.4/5 viruses with 100% accuracy. The assay can detect each genotype with a sensitivity as low as 39.1 copies per reaction. The intra- and inter-run precision are also satisfactory with CVs for the T_m_s ≤ 0.69% and ≤ 0.84%, as well as SDs ≤ 0.38 °C and ≤ 0.41 °C. The internal control (*ORF1ab* or *N*) in each reaction is detected to ensure reliability. The amplification products are directly analyzed in a closed-tube, which can avoid contamination caused by open tube. The design of probes and primers of this method are conventional and feasible, which can be generalized to novel mutations in the future. Also, this method has high-throughput potential for performance in a 96-well or 384-well PCR analyzer.

Moreover, the current melting curve method requires only one traditional probe for each mutation, which is cost-efficient compared to the other TaqMan-based real time PCR assays. TaqMan-MGB (minor groove binder)-PCR [27] and ARMS-PCR [25, 29, 30] are the two most commonly used TaqMan-based real time PCR assays for variant detection in clinical practice. TaqMan-MGB probe technology is optimized on the basis of the TaqMan probe, the 3' end of which is combined with a MGB to improve the accuracy and stability of hybridization by stabilizing the DNA double helix structure. And, the TaqMan-MGB probe combines a non-fluorescent quencher (NFQ) to greatly reduce the fluorescence background and improve sensitivity. The price of TaqMan-MGB probe is relatively more expensive than the ordinary TaqMan probe. Moreover, in TaqMan-MGB-PCR, two probes are required to identify the two different alleles of each variant. Thus, its cost of testing is at least twice of this melting curve assay. ARMS-PCR, also known as allele-specific PCR, requires different primer pairs to amplify each specific allele of a variant, combined with one TaqMan probe to identify the amplicons. Although only one ordinary TaqMan probe is needed, the precise genotyping of one variant has to be done in two reactions containing different specific primer pair to detect the alleles separately. Thus, the cost is also double compared to the present melting curve assay. In addition, the double-quenched probe [24] or LNA oligonucleotides [23] used in some TaqMan-based assays are even more expensive to make. Therefore, the melting curve-based assay has a great advantage in price, showing its potential to be applied to large-scale population screening.

For a multi-PCR assay, due to unequal reaction efficiencies for different amplicons or the interactions between oligonucleotide primers and probes, simultaneous amplification of multiple amplicons can be hampered, resulting in experimental failure and reduced analytical sensitivity [41, 42]. This problem is an even greater challenge for asymmetric PCR reactions, because the amplification rate is limited by the restriction primer. Meanwhile, the intensively distributed spike RBD mutations mediating the enhanced viral infectivity and immune escape of Omicron make it difficult to design separate primer pairs for each mutation. Also, adding more primer pairs into one assay leads to a higher likelihood of experimental failures. We successfully overcame these difficulties via the following approaches. First, as it has been previously recommended that amplification of specific spike protein regions that include the RBD is the most ideal for variant identification [17], we selected signature RBD mutations in the receptor-binding motif with functional significance. For spike fragment amplification, we designed a primer pair to amplify a 343-bp product, which covers all the mutations to be identified in this assay. Second, we adjusted and optimized the concentrations and proportions of primer pairs to amplify the spike, *ORF1ab*, and *N* fragments to ensure appropriate amplification efficiency for all products, while the combinations and concentrations of probes were precisely moderated. Finally, the PCR conditions were optimized to ensure the amplification of all fragments and that enough single-chain products were obtained efficiently, with PCR analysis done within 2.5 h.

As an PCR-based method, the current method cannot detect novel mutations, which is its major limitation. However, if an unexpected change of T_m_ is observed, it may prompt the occurrence of novel mutation in the probe targeted sequence, and sequencing methods should be then applied to clarify the exact mutation. Also, when there’s a need to apply this method to novel mutations, probes and primers should be reevaluated and usually redesigned. For assay design, to introduce as few amplification products as possible in the multiplex PCR is recommended, in order to reduce the risk of interference between the primer pairs. Longer amplicons can be employed when necessary, and the elongation process of each cycle should be extended accordingly. Notably, since the efficiency of asymmetric PCR is largely depended on the accurate concentration ratio of restriction primer and excessive primer, quantification of primers using a nucleic acid quantizer before experiment is recommended.

In addition, the presented method can be applied in a one-step RT-PCR system using viral RNA via adding random primer for reverse transcription, gene-specific primer pairs and probes in a commercial one-step PCR mixture and amplified directly. For the PCR procedure, a reverse transcription step (commonly 30 min) should be added before the initial denaturation step. With differences in components and their concentrations in the reaction buffer, the reference ranges of T_m_ for each melting peak may change slightly and should be determined preliminarily before use.

In conclusion, we described an asymmetric PCR-based melting curve analysis method for the rapid identification of immune-escape mutations located in the spike RBD of the SARS-CoV-2 Omicron strain. This assay can provide accurate, reliable, rapid, simple and low-cost preliminary detection of Omicron and its lineages in two PCR wells. We expect its use can be easily generalized in clinical laboratories with a fluorescent PCR platform.

### Supplementary Information

**Additional file 1**: **Table S1**. Insert sequence of plasmids.

**Additional file 2**: **Table S2**. Reaction mix composition for the asymmetric PCR melting curve analysis-based method.

**Additional file 3**: **Table S3**. Mean±SD values of T_m_s in single probe tests.

**Additional file 4**: **Fig. S1.** Performance of single probe tests. The performance of each single probe test for wild-type, Omicron BA.1, BA.2, BA.4/5, and BA2.75 with 312.5 template copies per reaction.

**Additional file 5**: **Fig. S2.** Limit of detection of single probe tests. The performance of each single test with varying amounts of plasmid templates at 1250, 625, 312.5, 156.3, 78.1, 39.1, 19.5, and 9.8 copies per reaction.

**Additional file 6**: **Fig. S3**. The asymmetric PCR melting curve analysis-based method clearly distinguishes Omicron variant and lineages in SARS-CoV-2-positive samples. The asymmetric PCR melting curve analysis-based method were performed in 40 SARS-CoV-2–positive clinical samples (including 36 BA.2 and 4 BA.4/5 samples) and pseudotyped lentiviruses of wild-type and BA.1 viral RNA control materials. (A) In reaction 1, four colored lines of each sample indicate the melting curves of RBD mutation site 460 (blue), 486 (green), 505 (orange) and the ORF1ab gene (red). (B) In reaction 2, the colored lines indicate the melting curves of RBD mutation site 484 (blue), 493+498 (green), 452 (orange) and the N gene (red).

**Additional file 7**: **Table S4**. Values of T_m_s in SARS-CoV-2-positive samples validation using the asymmetric PCR melting curve analysis-based method.

**Additional file 8**: **Table S5**. Sequence of the targeted spike gene region in SARS-CoV-2 positive clinical samples using Sanger sequencing.

## Data Availability

All data generated or analyzed during this study are included in this article and its additional files.

## Abbreviations

ACE2: Angiotensin-converting enzyme 2
ARMS: Amplification refractory mutation system
COVID-19: Coronavirus disease 2019
CV: Coefficient of variance
Flu A: Influenza virus A
Flu B: Influenza virus B
HRM: High-resolution melting
IP-RP-HPLC: Ion-pair reversed-phase high performance liquid chromatography
LNA: Locked nucleic acid
LOD: Limit of detection
MALDI-TOF MS: Matrix-assisted laser desorption ionization-time of flight mass spectrometry
MGB: Minor groove binder
NGS: Next-generation sequencing
PCR: Polymerase chain reaction
RBD: Receptor-binding domain
RSV: Respiratory syncytial virus
Rt: Effective reproduction number
RT-PCR: Reverse-transcription polymerase chain reaction
SARS-CoV-2: Severe acute respiratory syndrome-coronavirus-2
SD: Standard deviation
T_m_: Melting temperature
VOC: Variant of concern
WGS: Whole-genome sequencing
WHO: World Health Organization
