# Supplementary material for: Discrimination of SARS-CoV-2 omicron variant and its lineages by rapid detection of immune-escape mutations in spike protein RBD using asymmetric PCR-based melting curve analysis
Source: Virol J. 2023 Aug 25;20:192. doi: 10.1186/s12985-023-02137-5 (PMC10463914; doi:10.1186/s12985-023-02137-5)
Supplement: Supplementary file 1 — Additional file 1: Table S1. Insert sequence of plasmids. [file 12985_2023_2137_MOESM1_ESM.docx]

**Table S1.** Insert sequence of plasmids.

| **Plasmid** | **Insert sequence (5'→3')** | **Fragment size (bp)** |
| --- | --- | --- |
| **Wuhan_S_1212-1584** | TGATGAAGTCAGACAAATCGCTCCAGGGCAAACTGGAAAGATTGCTGATTATAATTATAAATTACCAGATGATTTTACAGGCTGCGTTATAGCTTGGAATTCTAACAATCTTGATTCTAAGGTTGGTGGTAATTATAATTACCTGTATAGATTGTTTAGGAAGTCTAATCTCAAACCTTTTGAGAGAGATATTTCAACTGAAATCTATCAGGCCGGTAGCACACCTTGTAATGGTGTTGAAGGTTTTAATTGTTACTTTCCTTTACAATCATATGGTTTCCAACCCACTAATGGTGTTGGTTACCAACCATACAGAGTAGTAGTACTTTCTTTTGAACTTCTACATGCACCAGCAACTGTTTGTGGACCTAAA | 373 |
| **BA.1.1.2_S_1129-1584** | TTTAAGTGTTATGGAGTGTCTCCTACTAAATTAAATGATCTCTGCTTTACTAATGTCTATGCAGATTCATTTGTAATTAGAGGTGATGAAGTCAGACAAATCGCTCCAGGGCAAACTGGAAATATTGCTGATTATAATTATAAATTACCAGATGATTTTACAGGCTGCGTTATAGCTTGGAATTCTAACAAGCTTGATTCTAAGGTTAGTGGTAATTATAATTACCTGTATAGATTGTTTAGGAAGTCTAATCTCAAACCTTTTGAGAGAGATATTTCAACTGAAATCTATCAGGCCGGTAACAAACCTTGTAATGGTGTTGCAGGTTTTAATTGTTACTTTCCTTTACGATCATATAGTTTCCGACCCACTTATGGTGTTGGTCACCAACCATACAGAGTAGTAGTACTTTCTTTTGAACTTCTACATGCACCAGCAACTGTTTGTGGACCTAAA | 456 |
| **BA.2_S_1212-1584** | TAATGAAGTCAGCCAAATCGCTCCAGGGCAAACTGGAAATATTGCTGATTATAATTATAAATTACCAGATGATTTTACAGGCTGCGTTATAGCTTGGAATTCTAACAAGCTTGATTCTAAGGTTGGTGGTAATTATAATTACCTGTATAGATTGTTTAGGAAGTCTAATCTCAAACCTTTTGAGAGAGATATTTCAACTGAAATCTATCAGGCCGGTAACAAACCTTGTAATGGTGTTGCAGGTTTTAATTGTTACTTTCCTTTACGATCATATGGTTTCCGACCCACTTATGGTGTTGGTCACCAACCATACAGAGTAGTAGTACTTTCTTTTGAACTTCTACATGCACCAGCAACTGTTTGTGGACCTAAA | 373 |
| **BA.4/BA.5_S_1129-1584** | TTTAAGTGTTATGGAGTGTCTCCTACTAAATTAAATGATCTCTGCTTTACTAATGTCTATGCAGATTCATTTGTAATTAGAGGTAATGAAGTCAGCCAAATCGCTCCAGGGCAAACTGGAAATATTGCTGATTATAATTATAAATTACCAGATGATTTTACAGGCTGCGTTATAGCTTGGAATTCTAACAAGCTTGATTCTAAGGTTGGTGGTAATTATAATTACCGGTATAGATTGTTTAGGAAGTCTAATCTCAAACCTTTTGAGAGAGATATTTCAACTGAAATCTATCAGGCCGGTAACAAACCTTGTAATGGTGTTGCAGGTGTTAATTGTTACTTTCCTTTACAATCATATGGTTTCCGACCCACTTATGGTGTTGGTCACCAACCATACAGAGTAGTAGTACTTTCTTTTGAACTTCTACATGCACCAGCAACTGTTTGTGGACCTAAA | 456 |
| **BA2.75_S_1129-1584** | TTTAAGTGTTATGGAGTGTCTCCTACTAAATTAAATGATCTCTGCTTTACTAATGTCTATGCAGATTCATTTGTAATTAGAGGTAATGAAGTCAGCCAAATCGCTCCAGGGCAAACTGGAAATATTGCTGATTATAATTATAAATTACCAGATGATTTTACAGGCTGCGTTATAGCTTGGAATTCTAACAAGCTTGATTCTAAGGTTAGTGGTAATTATAATTACCTGTATAGATTGTTTAGGAAGTCTAAGCTCAAACCTTTTGAGAGAGATATTTCAACTGAAATCTATCAGGCCGGTAACAAACCTTGTAATGGTGTTGCAGGTTTTAATTGTTACTTTCCTTTACAATCATATGGTTTCCGACCCACTTATGGTGTTGGTCACCAACCATACAGAGTAGTAGTACTTTCTTTTGAACTTCTACATGCACCAGCAACTGTTTGTGGACCTAAA | 456 |
| **N_481-830** | CTTCCTCAAGGAACAACATTGCCAAAAGGCTTCTACGCAGAAGGGAGCAGAGGCGGCAGTCAAGCCTCTTCTCGTTCCTCATCACGTAGTCGCAACAGTTCAAGAAATTCAACTCCAGGCAGCAGTAGGGGAACTTCTCCTGCTAGAATGGCTGGCAATGGCGGTGATGCTGCTCTTGCTTTGCTGCTGCTTGACAGATTGAACCAGCTTGAGAGCAAAATGTCTGGTAAAGGCCAACAACAACAAGGCCAAACTGTCACTAAGAAATCTGCTGCTGAGGCTTCTAAGAAGCCTCGGCAAAAACGTACTGCCACTAAAGCATACAATGTAACACAAGCTTTCGGCAGACG | 350 |
| **ORF1ab_12961-13310** | TTTGGTGGTGCATCGTGTTGTCTGTACTGCCGTTGCCACATAGATCATCCAAATCCTAAAGGATTTTGTGACTTAAAAGGTAAGTATGTACAAATACCTACAACTTGTGCTAATGACCCTGTGGGTTTTACACTTAAAAACACAGTCTGTACCGTCTGCGGTATGTGGAAAGGTTATGGCTGTAGTTGTGATCAACTCCGCGAACCCATGCTTCAGTCAGCTGATGCACAATCGTTTTTAAACGGGTTTGCGGTGTAAGTGCAGCCCGTCTTACACCGTGCGGCACAGGCACTAGTACTGATGTCGTATACAGGGCTTTTGACATCTACAATGATAAAGTAGCTGGTTTT | 350 |
