# Supplementary material for: Discrimination of SARS-CoV-2 omicron variant and its lineages by rapid detection of immune-escape mutations in spike protein RBD using asymmetric PCR-based melting curve analysis
Source: Virol J. 2023 Aug 25;20:192. doi: 10.1186/s12985-023-02137-5 (PMC10463914; doi:10.1186/s12985-023-02137-5)
Supplement: Supplementary file 2 — Additional file 2: Table S2. Reaction mix composition for the asymmetric PCR melting curve analysis-based method. [file 12985_2023_2137_MOESM2_ESM.docx]

**Table S2.** Reaction mix composition for the asymmetric PCR melting curve analysis-based method.

| **Reaction 1** | | **Reaction 2** | |
| --- | --- | --- | --- |
| **Component** | **Final Concentration** |  | **Final Concentration** |
| TAKARA Ex Taq Buffer | 1× | TAKARA Ex Taq Buffer | 1× |
| Mg2^+^ | 2 mM | Mg2^+^ | 2 mM |
| dNTP | 0.2 mM of each dNTP | dNTP | 0.2 mM of each dNTP |
| *ORF1ab* gene forward primer | 0.3 μM | *N* gene forward primer | 0.1 μM |
| *ORF1ab* gene reverse primer | 2.4 μM | *N* gene reverse primer | 0.8 μM |
| *ORF1ab*-Cy5 probe | 0.4 μM | *N*-Cy5 probe | 0.2 μM |
| Spike gene forward primer | 0.1 μM | Spike gene forward primer | 0.2 μM |
| Spike gene reverse primer | 0.8 μM | Spike gene reverse primer | 1.6 μM |
| 460K-FAM probe | 0.2 μM | 484A-FAM probe | 0.1 μM |
| 486V-HEX probe | 0.2 μM | 493R+498R-HEX probe | 0.4 μM |
| 505H-ROX probe | 0.1 μM | 452R-ROX probe | 0.1 μM |
| TAKARA Ex Taq DNA polymerase | 0.625 U | TAKARA Ex Taq DNA polymerase | 0.625 U |
