# Supplementary material for: Discrimination of SARS-CoV-2 omicron variant and its lineages by rapid detection of immune-escape mutations in spike protein RBD using asymmetric PCR-based melting curve analysis
Source: Virol J. 2023 Aug 25;20:192. doi: 10.1186/s12985-023-02137-5 (PMC10463914; doi:10.1186/s12985-023-02137-5)
Supplement: Supplementary file 3 — Additional file 3: Table S3. Mean±SD values of Tms in single probe tests. [file 12985_2023_2137_MOESM3_ESM.docx]

**Table S3.** Mean±SD values of T_m_s in single probe tests.

| **Sequence** | **Mean±SD, ℃** | | | | | | | |
| --- | --- | --- | --- | --- | --- | --- | --- | --- |
|  | **Probe-460K-FAM** | **Probe-486V-HEX** | **Probe-505H-ROX** | **Probe-*Orf1ab*-Cy5** | **Probe-484A-FAM** | **Probe-493R+498R-HEX** | **Probe-452R-ROX** | **Probe-*N*-Cy5** |
| **Wild type** | 58.59±0.17 | 47.10±0.07 | 52.76±0.06 | 64.92±0.17 | 46.13±0.11 | 49.30±0.07 | 50.78±0.05 | 63.95±0.19 |
| **BA.1** | 58.68±0.08 | 47.04±0.07 | 57.16±0.13 | 64.92±0.14 | 58.54±0.10 | 53.57±0.22 | 50.85±0.09 | 63.96±0.14 |
| **BA.2** | 58.78±0.10 | 47.15±0.10 | 57.14±0.07 | 64.91±0.13 | 58.66±0.12 | 59.79±0.30 | 50.76±0.08 | 63.95±0.07 |
| **BA.4/5** | 58.72±0.08 | 54.86±0.06 | 57.07±0.04 | 64.90±0.13 | 53.04±0.09 | 55.47±0.34 | 57.43±0.06 | 63.96±0.10 |
| **BA.2.75** | 63.16±0.16 | 47.11±0.08 | 57.14±0.11 | 64.93±0.19 | 58.65±0.10 | 55.35±0.14 | 50.77±0.07 | 63.97±0.12 |
