# Supplementary figures and images for: Discrimination of SARS-CoV-2 omicron variant and its lineages by rapid detection of immune-escape mutations in spike protein RBD using asymmetric PCR-based melting curve analysis
Source: Virol J. 2023 Aug 25;20:192. doi: 10.1186/s12985-023-02137-5 (PMC10463914; doi:10.1186/s12985-023-02137-5)

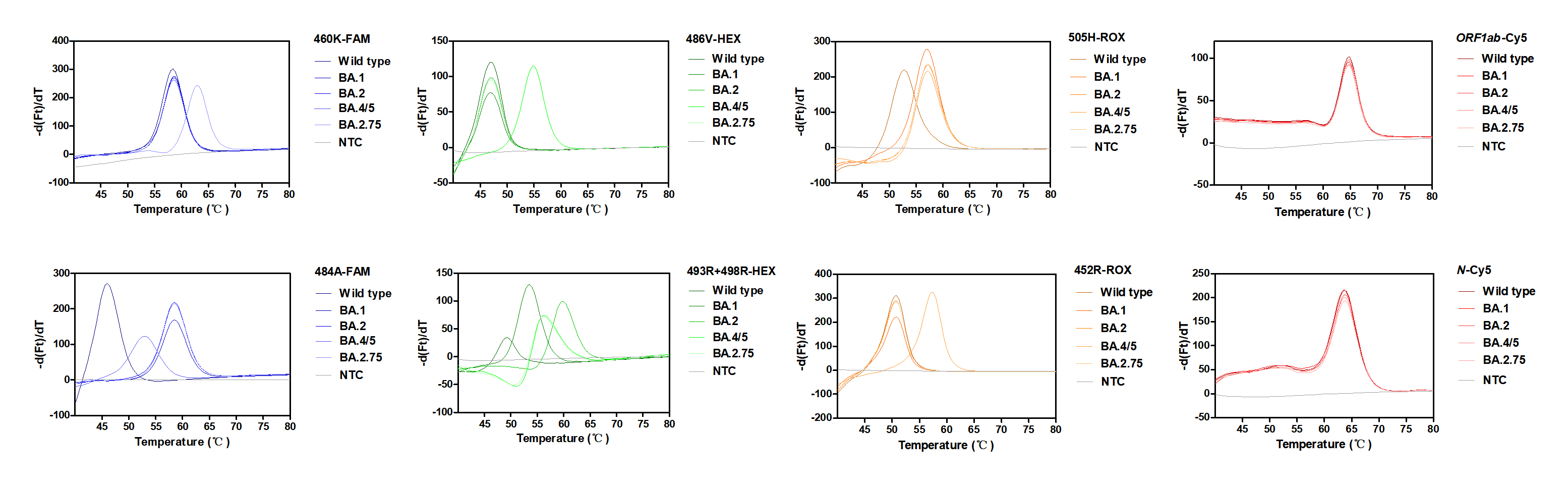

Supplement: Supplementary file 4 — Additional file 4: Fig. S1. Performance of single probe tests. The performance of each single probe test for wild-type, Omicron BA.1, BA.2, BA.4/5, and BA2.75 with 312.5 template copies per reaction. [file 12985_2023_2137_MOESM4_ESM.jpg]

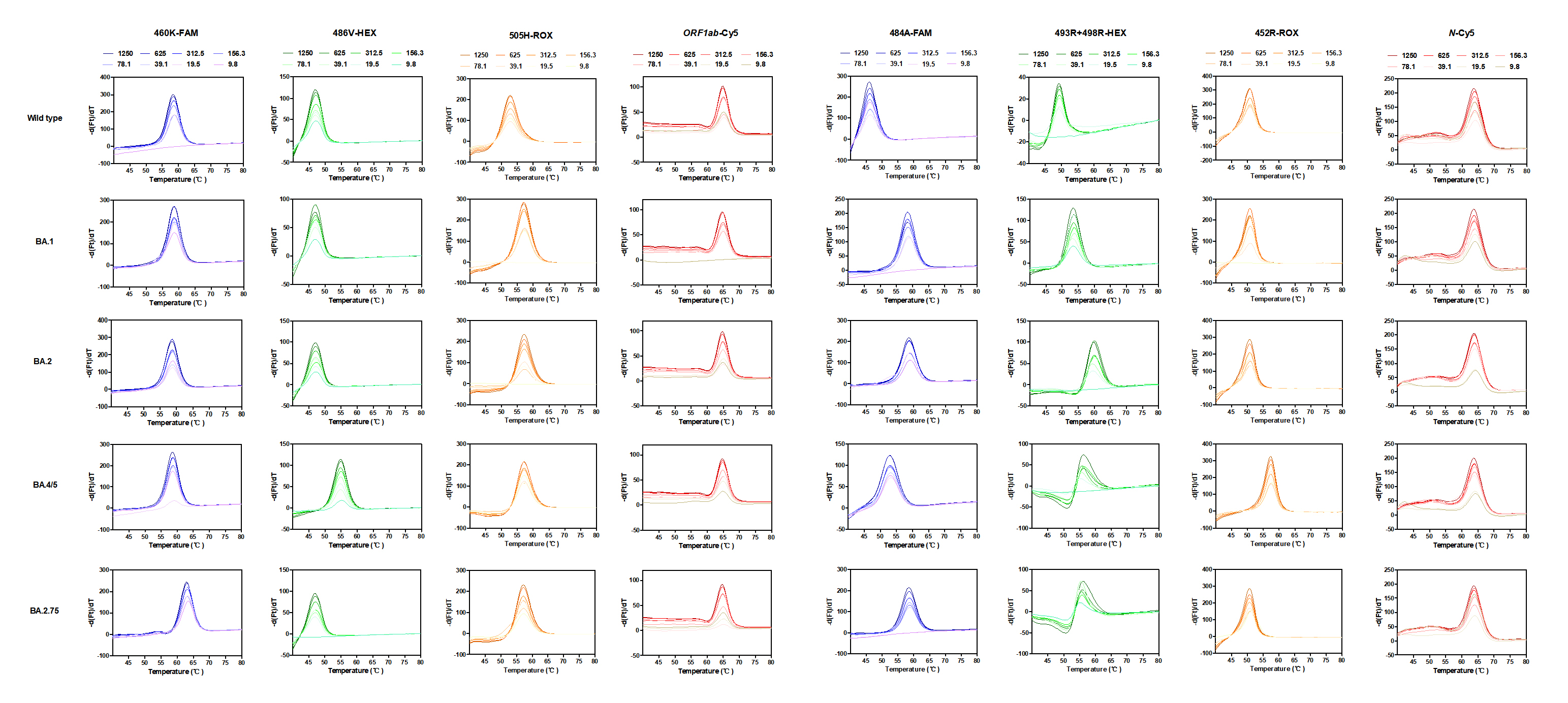

Supplement: Supplementary file 5 — Additional file 5: Fig. S2. Limit of detection of single probe tests. The performance of each single test with varying amounts of plasmid templates at 1250, 625, 312.5, 156.3, 78.1, 39.1, 19.5, and 9.8 copies per reaction. [file 12985_2023_2137_MOESM5_ESM.jpg]

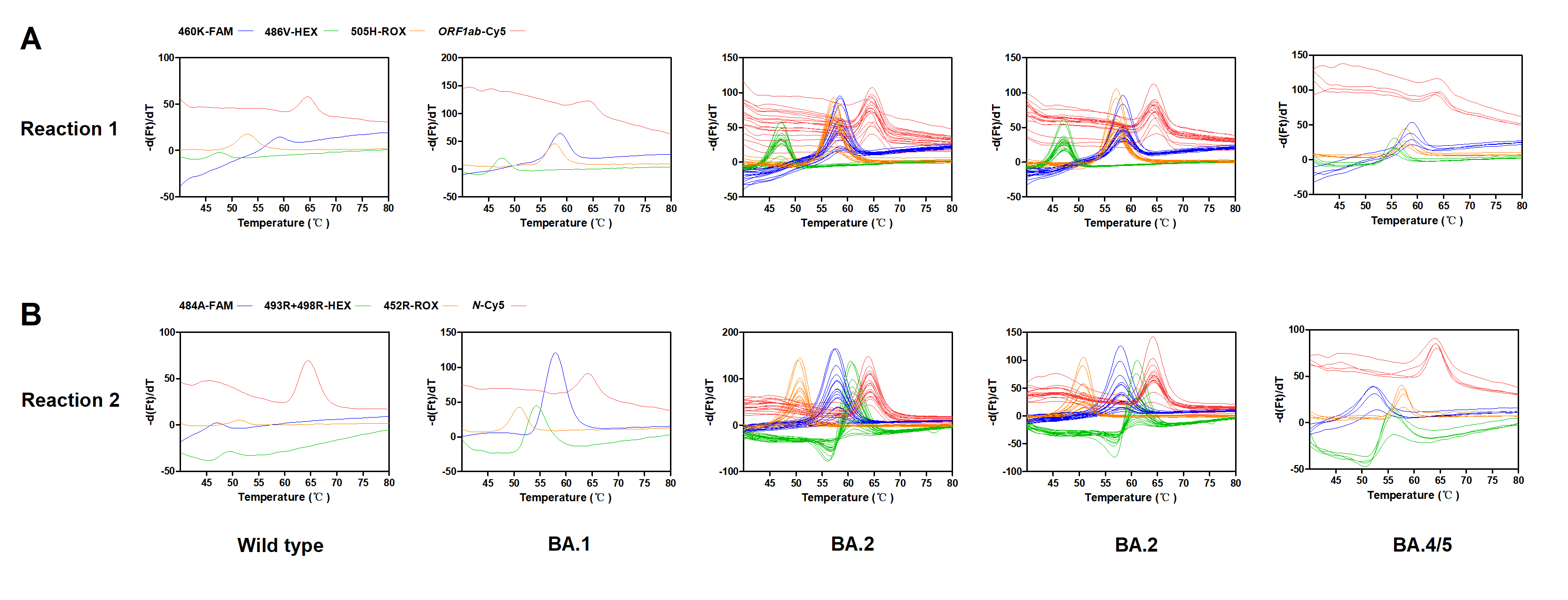

Supplement: Supplementary file 6 — Additional file 6: Fig. S3. The asymmetric PCR melting curve analysis-based method clearly distinguishes Omicron variant and lineages in SARS-CoV-2-positive samples. The asymmetric PCR melting curve analysis-based method were performed in 40 SARS-CoV-2–positive clinical samples (including 36 BA.2 and 4 BA.4/5 samples) and pseudotyped lentiviruses of wild-type and BA.1 viral RNA control materials. (A) In reaction 1, four colored lines of each sample indicate the melting curves of RBD mutation site 460 (blue), 486 (green), 505 (orange) and the ORF1ab gene (red). (B) In reaction 2, the colored lines indicate the melting curves of RBD mutation site 484 (blue), 493+498 (green), 452 (orange) and the N gene (red). [file 12985_2023_2137_MOESM6_ESM.jpg]
