# Supplementary material for: Discrimination of SARS-CoV-2 omicron variant and its lineages by rapid detection of immune-escape mutations in spike protein RBD using asymmetric PCR-based melting curve analysis
Source: Virol J. 2023 Aug 25;20:192. doi: 10.1186/s12985-023-02137-5 (PMC10463914; doi:10.1186/s12985-023-02137-5)
Supplement: Supplementary file 7 — Additional file 7: Table S4. Values of Tms in SARS-CoV-2-positive samples validation using the asymmetric PCR melting curve analysis-based method. [file 12985_2023_2137_MOESM7_ESM.docx]

**Table S4**. Values of T_m_s in SARS-CoV-2-positive samples validation using the asymmetric PCR melting curve analysis-based method.

| **ID** | **Lineage** | **Reaction 1** | | | | | | | **Reaction 2** | | | | | | |
| --- | --- | --- | --- | --- | --- | --- | --- | --- | --- | --- | --- | --- | --- | --- | --- |
|  |  | **Probe-460K-FAM** | | **Probe-486V-HEX** | | **Probe-505H-ROX** | | **Probe-*Orf1ab*-Cy5** | **Probe-484A-FAM** | | **Probe-493R+498R-HEX** | | **Probe-452R-ROX** | | **Probe-*N*-Cy5** |
|  |  | **Genotype** | **T_m_, ℃** | **Genotype** | **T_m_, ℃** | **Genotype** | **T_m_, ℃** | **T_m_, ℃** | **Genotype** | **T_m_, ℃** | **Genotype** | **T_m_, ℃** | **Genotype** | **T_m_, ℃** | **T_m_, ℃** |
| **SARS-CoV-2 RNA weak positive quality control reference material** | Wild type | 460N | 59.01 | 486F | 47.58 | 505Y | 53.00 | 64.62 | 484E, 486F | 46.90 | 493Q, 496G, 498Q | 49.06 | 452L | 51.44 | 64.39 |
| **SARS-CoV-2 Omicron BA.1 quality control reference material** | BA.1 | 460N | 58.66 | 486F | 47.46 | 505H | 57.59 | 64.66 | 484A, 486F | 57.88 | 493R, 496S, 498R | 54.10 | 452L | 51.07 | 64.29 |
| **Ba2** | BA.2 | 460N | 58.85 | 486F | 47.65 | 505H | 57.48 | 65.02 | 484A, 486F | 58.06 | 493R, 496G, 498R | 60.79 | 452L | 50.90 | 64.29 |
| **M163** | BA.4/5 | 460N | 58.75 | 486V | 55.44 | 505H | 57.60 | 63.83 | 484A, 486V | 52.55 | 493Q, 496G, 498R | 55.25 | 452R | 57.77 | 64.20 |
| **412** | BA.2 | 460N | 58.43 | 486F | 47.36 | 505H | 57.27 | 64.72 | 484A, 486F | 57.87 | 493R, 496G, 498R | 60.54 | 452L | 50.77 | 64.06 |
| **518** | BA.2 | 460N | 58.61 | 486F | 47.62 | 505H | 57.43 | 64.87 | 484A, 486F | 58.23 | 493R, 496G, 498R | 60.80 | 452L | 51.06 | 64.16 |
| **523** | BA.2 | 460N | 58.38 | 486F | 47.42 | 505H | 57.45 | 64.08 | 484A, 486F | 58.43 | 493R, 496G, 498R | 60.85 | 452L | 50.88 | 64.25 |
| **554** | BA.2 | 460N | 58.57 | 486F | 47.28 | 505H | 57.23 | 64.11 | 484A, 486F | 58.13 | 493R, 496G, 498R | 60.90 | 452L | 50.96 | 64.24 |
| **561** | BA.2 | 460N | 58.66 | 486F | 47.37 | 505H | 57.30 | 64.46 | 484A, 486F | 58.11 | 493R, 496G, 498R | 60.90 | 452L | 50.94 | 64.23 |
| **573** | BA.2 | 460N | 58.56 | 486F | 47.37 | 505H | 57.36 | 64.74 | 484A, 486F | 57.78 | 493R, 496G, 498R | 60.48 | 452L | 50.84 | 64.04 |
| **577** | BA.2 | 460N | 58.52 | 486F | 47.36 | 505H | 57.28 | 64.53 | 484A, 486F | 58.14 | 493R, 496G, 498R | 60.73 | 452L | 50.9 | 64.21 |
| **579** | BA.2 | 460N | 58.63 | 486F | 47.32 | 505H | 57.28 | 64.45 | 484A, 486F | 57.96 | 493R, 496G, 498R | 60.63 | 452L | 50.79 | 64.14 |
| **69** | BA.2 | 460N | 58.61 | 486F | 47.48 | 505H | 57.34 | 64.47 | 484A, 486F | 57.91 | 493R, 496G, 498R | 60.59 | 452L | 50.93 | 64.13 |
| **618** | BA.2 | 460N | 58.67 | 486F | 47.44 | 505H | 57.41 | 64.68 | 484A, 486F | 57.67 | 493R, 496G, 498R | 60.44 | 452L | 50.87 | 64.12 |
| **649** | BA.2 | 460N | 58.76 | 486F | 47.50 | 505H | 57.49 | 64.54 | 484A, 486F | 57.96 | 493R, 496G, 498R | 60.60 | 452L | 50.95 | 64.15 |
| **M34** | BA.4/5 | 460N | 58.51 | 486V | 55.28 | 505H | 57.54 | 64.41 | 484A, 486V | 52.19 | 493Q, 496G, 498R | 55.18 | 452R | 57.61 | 64.20 |
| **580** | BA.2 | 460N | 58.56 | 486F | 47.27 | 505H | 57.08 | 64.63 | 484A, 486F | 57.99 | 493R, 496G, 498R | 61.22 | 452L | 50.79 | 64.01 |
| **584** | BA.2 | 460N | 58.44 | 486F | 47.23 | 505H | 57.24 | 64.69 | 484A, 486F | 57.93 | 493R, 496G, 498R | 60.64 | 452L | 50.87 | 64.19 |
| **585** | BA.2 | 460N | 58.61 | 486F | 47.33 | 505H | 57.22 | 64.79 | 484A, 486F | 58.18 | 493R, 496G, 498R | 60.88 | 452L | 50.94 | 64.21 |
| **592** | BA.2 | 460N | 58.66 | 486F | 47.32 | 505H | 57.26 | 64.71 | 484A, 486F | 58.17 | 493R, 496G, 498R | 60.88 | 452L | 50.85 | 64.16 |
| **661** | BA.2 | 460N | 58.69 | 486F | 47.51 | 505H | 57.43 | 64.41 | 484A, 486F | 57.95 | 493R, 496G, 498R | 60.68 | 452L | 50.86 | 64.15 |
| **673** | BA.2 | 460N | 58.64 | 486F | 47.49 | 505H | 57.38 | 64.59 | 484A, 486F | 57.80 | 493R, 496G, 498R | 60.37 | 452L | 50.84 | 64.07 |
| **680** | BA.2 | 460N | 58.77 | 486F | 47.59 | 505H | 57.37 | 64.69 | 484A, 486F | 58.05 | 493R, 496G, 498R | 60.73 | 452L | 50.89 | 64.1 |
| **682** | BA.2 | 460N | 58.53 | 486F | 47.34 | 505H | 57.33 | 64.45 | 484A, 486F | 57.36 | 493R, 496G, 498R | 60.11 | 452L | 50.53 | 63.84 |
| **760** | BA.2 | 460N | 58.47 | 486F | 47.21 | 505H | 57.24 | 64.57 | 484A, 486F | 58.06 | 493R, 496G, 498R | 60.63 | 452L | 50.92 | 64.2 |
| **765** | BA.2 | 460N | 58.47 | 486F | 47.28 | 505H | 57.25 | 64.44 | 484A, 486F | 58.06 | 493R, 496G, 498R | 60.70 | 452L | 50.91 | 64.15 |
| **784** | BA.2 | 460N | 58.60 | 486F | 47.42 | 505H | 57.33 | 63.59 | 484A, 486F | 58.42 | 493R, 496G, 498R | 61.22 | 452L | 50.9 | 64.29 |
| **789** | BA.2 | 460N | 58.53 | 486F | 47.39 | 505H | 57.40 | 64.75 | 484A, 486F | 58.16 | 493R, 496G, 498R | 60.81 | 452L | 51.03 | 64.33 |
| **816** | BA.2 | 460N | 58.58 | 486F | 47.26 | 505H | 57.29 | 64.08 | 484A, 486F | 58.14 | 493R, 496G, 498R | 60.60 | 452L | 51.02 | 64.22 |
| **860** | BA.2 | 460N | 58.60 | 486F | 47.35 | 505H | 57.36 | 64.52 | 484A, 486F | 58.15 | 493R, 496G, 498R | 60.80 | 452L | 51.08 | 64.28 |
| **873** | BA.2 | 460N | 58.59 | 486F | 47.40 | 505H | 57.26 | 65.01 | 484A, 486F | 58.29 | 493R, 496G, 498R | 61.17 | 452L | 51.17 | 63.75 |
| **877** | BA.2 | 460N | 58.48 | 486F | 47.20 | 505H | 57.21 | 64.59 | 484A, 486F | 58.11 | 493R, 496G, 498R | 61.06 | 452L | 51.13 | 64.35 |
| **884** | BA.2 | 460N | 58.40 | 486F | 47.23 | 505H | 57.23 | 64.62 | 484A, 486F | 57.87 | 493R, 496G, 498R | 60.56 | 452L | 50.77 | 64.09 |
| **885** | BA.2 | 460N | 58.50 | 486F | 47.26 | 505H | 57.18 | 63.85 | 484A, 486F | 57.89 | 493R, 496G, 498R | 60.61 | 452L | 50.81 | 64.07 |
| **93** | BA.2 | 460N | 58.53 | 486F | 47.43 | 505H | 57.56 | 64.79 | 484A, 486F | 58.13 | 493R, 496G, 498R | 60.82 | 452L | 51.07 | 64.22 |
| **912** | BA.2 | 460N | 58.53 | 486F | 47.56 | 505H | 57.49 | 64.88 | 484A, 486F | 58.11 | 493R, 496G, 498R | 60.76 | 452L | 51.06 | 64.28 |
| **913** | BA.2 | 460N | 58.43 | 486F | 47.30 | 505H | 57.28 | 64.50 | 484A, 486F | 58.03 | 493R, 496G, 498R | 60.72 | 452L | 50.98 | 64.18 |
| **949** | BA.2 | 460N | 58.56 | 486F | 47.34 | 505H | 57.34 | 64.49 | 484A, 486F | 58.17 | 493R, 496G, 498R | 60.82 | 452L | 51.11 | 64.3 |
| **961** | BA.2 | 460N | 58.59 | 486F | 47.44 | 505H | 57.29 | 64.66 | 484A, 486F | 58.27 | 493R, 496G, 498R | 60.87 | 452L | 51.14 | 64.3 |
| **984** | BA.2 | 460N | 58.49 | 486F | 47.20 | 505H | 57.18 | 64.44 | 484A, 486F | 57.94 | 493R, 496G, 498R | 60.74 | 452L | 50.92 | 64.09 |
| **M90** | BA.4/5 | 460N | 58.92 | 486V | 55.47 | 505H | 57.59 | 64.09 | 484A, 486V | 52.76 | 493Q, 496G, 498R | 55.37 | 452R | 57.87 | 64.4 |
| **M84** | BA.4/5 | 460N | 58.57 | 486V | 55.50 | 505H | 57.83 | 64.46 | 484A, 486V | 52.45 | 493Q, 496G, 498R | 55.41 | 452R | 57.96 | 64.19 |
