# Supplementary material for: Discrimination of SARS-CoV-2 omicron variant and its lineages by rapid detection of immune-escape mutations in spike protein RBD using asymmetric PCR-based melting curve analysis
Source: Virol J. 2023 Aug 25;20:192. doi: 10.1186/s12985-023-02137-5 (PMC10463914; doi:10.1186/s12985-023-02137-5)
Supplement: Supplementary file 8 — Additional file 8: Table S5. Sequence of the targeted spike gene region in SARS-CoV-2 positive clinical samples using Sanger sequencing. [file 12985_2023_2137_MOESM8_ESM.docx]

**Table S5**. Sequence of the targeted spike gene region in SARS-CoV-2 positive clinical samples using Sanger sequencing.

| **ID** | **Lineage** | **Sequence of the targeted region in spike gene**  **(Amino acids 427-518)** | | |
| --- | --- | --- | --- | --- |
| Ba2 | BA.2 | GATGATTTTACAGGCTGCGTTATAGCTTGGAATTCTAACAAGCTTGATTCTAAGGTTGGTGGTAATTATAATTACCTGTATAGATTGTTTAGGAAGTCTAATCTCAAACCTTTTGAGAGAGATATTTCAACTGAAATCTATCAGGCCGGTAACAAACCTTGTAATGGTGTTGCAGGTTTTAATTGTTACTTTCCTTTACGATCATATGGTTTCCGACCCACTTATGGTGTTGGTCACCAACCATACAGAGTAGTAGTACTTTCTTTTGAACTTCTA | | |
|  |  | **Genotype** | **Codon** | **Sanger sequencing diagram** |
|  |  | 452L | CTG | 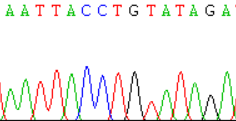 |
|  |  | 460N | AAT | 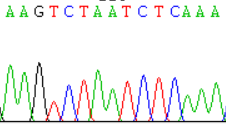 |
|  |  | 484A, 486F | GCA, TTT | 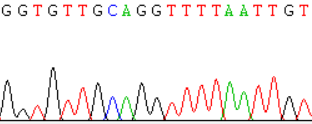 |
|  |  | 493R | CGA | 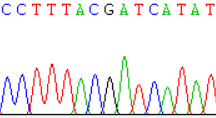 |
|  |  | 498R | CGA | 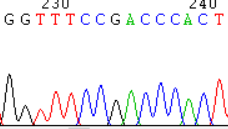 |
|  |  | 505H | CAC | 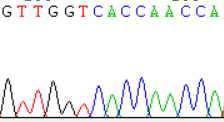 |
| M163 | BA.4/5 | GATGATTTTACAGGCTGCGTTATAGCTTGGAATTCTAACAAGCTTGATTCTAAGGTTGGTGGTAATTATAATTACCGGTATAGATTGTTTAGGAAGTCTAATCTCAAACCTTTTGAGAGAGATATTTCAACTGAAATCTATCAGGCCGGTAACAAACCTTGTAATGGTGTTGCAGGTGTTAATTGTTACTTTCCTTTACAATCATATGGTTTCCGACCCACTTATGGTGTTGGTCACCAACCATACAGAGTAGTAGTACTTTCTTTTGAACTTCTA | | |
|  |  | **Genotype** | **Codon** | **Sanger sequencing diagram** |
|  |  | 452R | CGG | 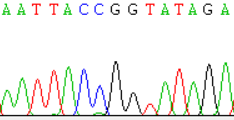 |
|  |  | 460N | AAT | 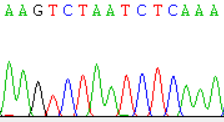 |
|  |  | 484A, 486V | GCA, GTT | 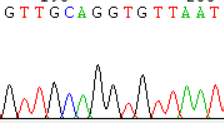 |
|  |  | 493Q | CAA | 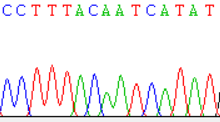 |
|  |  | 498R | CGA | 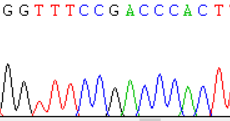 |
|  |  | 505H | CAC | 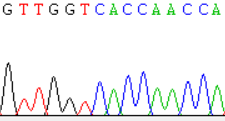 |
| 412 | BA.2 | GATGATTTTACAGGCTGCGTTATAGCTTGGAATTCTAACAAGCTTGATTCTAAGGTTGGTGGTAATTATAATTACCTGTATAGATTGTTTAGGAAGTCTAATCTCAAACCTTTTGAGAGAGATATTTCAACTGAAATCTATCAGGCCGGTAACAAACCTTGTAATGGTGTTGCAGGTTTTAATTGTTACTTTCCTTTACGATCATATGGTTTCCGACCCACTTATGGTGTTGGTCACCAACCATACAGAGTAGTAGTACTTTCTTTTGAACTTCTA | | |
|  |  | **Genotype** | **Codon** | **Sanger sequencing diagram** |
|  |  | 452L | CTG | 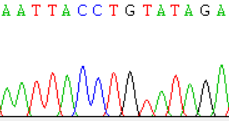 |
|  |  | 460N | AAT | 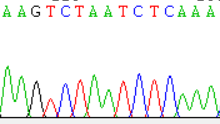 |
|  |  | 484A, 486F | GCA, TTT | 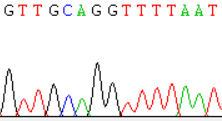 |
|  |  | 493R | CGA | 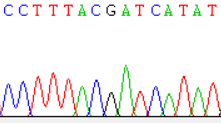 |
|  |  | 498R | CGA | 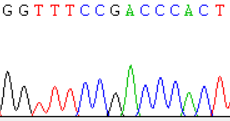 |
|  |  | 505H | CAC | 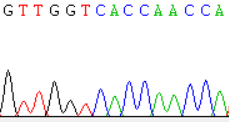 |
| 518 | BA.2 | GATGATTTTACAGGCTGCGTTATAGCTTGGAATTCTAACAAGCTTGATTCTAAGGTTGGTGGTAATTATAATTACCTGTATAGATTGTTTAGGAAGTCTAATCTCAAACCTTTTGAGAGAGATATTTCAACTGAAATCTATCAGGCCGGTAACAAACCTTGTAATGGTGTTGCAGGTTTTAATTGTTACTTTCCTTTACGATCATATGGTTTCCGACCCACTTATGGTGTTGGTCACCAACCATACAGAGTAGTAGTACTTTCTTTTGAACTTCTA | | |
|  |  | **Genotype** | **Codon** | **Sanger sequencing diagram** |
|  |  | 452L | CTG | 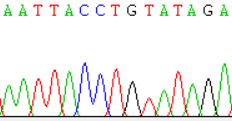 |
|  |  | 460N | AAT | 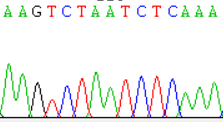 |
|  |  | 484A, 486F | GCA, TTT | 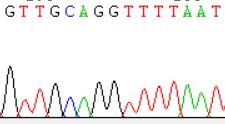 |
|  |  | 493R | CGA | 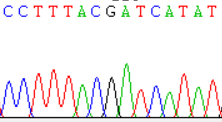 |
|  |  | 498R | CGA | 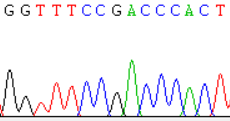 |
|  |  | 505H | CAC | 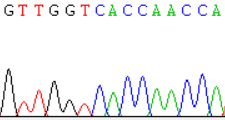 |
| 523 | BA.2 | GATGATTTTACAGGCTGCGTTATAGCTTGGAATTCTAACAAGCTTGATTCTAAGGTTGGTGGTAATTATAATTACCTGTATAGATTGTTTAGGAAGTCTAATCTCAAACCTTTTGAGAGAGATATTTCAACTGAAATCTATCAGGCCGGTAACAAACCTTGTAATGGTGTTGCAGGTTTTAATTGTTACTTTCCTTTACGATCATATGGTTTCCGACCCACTTATGGTGTTGGTCACCAACCATACAGAGTAGTAGTACTTTCTTTTGAACTTCTA | | |
|  |  | **Genotype** | **Codon** | **Sanger sequencing diagram** |
|  |  | 452L | CTG | 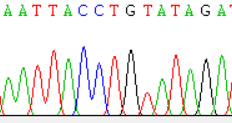 |
|  |  | 460N | AAT | 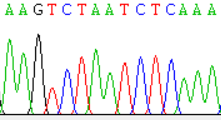 |
|  |  | 484A, 486F | GCA, TTT | 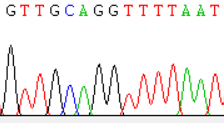 |
|  |  | 493R | CGA | 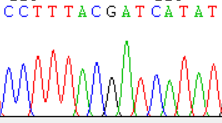 |
|  |  | 498R | CGA | 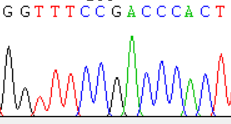 |
|  |  | 505H | CAC | 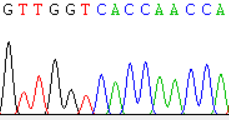 |
| 554 | BA.2 | GATGATTTTACAGGCTGCGTTATAGCTTGGAATTCTAACAAGCTTGATTCTAAGGTTGGTGGTAATTATAATTACCTGTATAGATTGTTTAGGAAGTCTAATCTCAAACCTTTTGAGAGAGATATTTCAACTGAAATCTATCAGGCCGGTAACAAACCTTGTAATGGTGTTGCAGGTTTTAATTGTTACTTTCCTTTACGATCATATGGTTTCCGACCCACTTATGGTGTTGGTCACCAACCATACAGAGTAGTAGTACTTTCTTTTGAACTTCTA | | |
|  |  | **Genotype** | **Codon** | **Sanger sequencing diagram** |
|  |  | 452L | CTG | 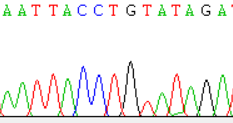 |
|  |  | 460N | AAT | 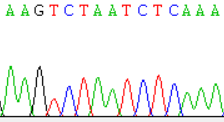 |
|  |  | 484A, 486F | GCA, TTT | 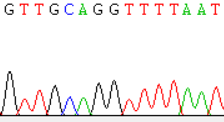 |
|  |  | 493R | CGA | 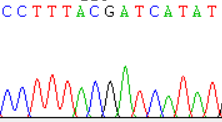 |
|  |  | 498R | CGA | 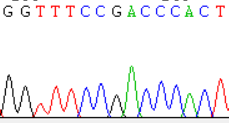 |
|  |  | 505H | CAC | 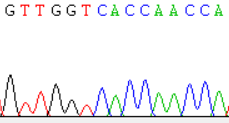 |
| 561 | BA.2 | GATGATTTTACAGGCTGCGTTATAGCTTGGAATTCTAACAAGCTTGATTCTAAGGTTGGTGGTAATTATAATTACCTGTATAGATTGTTTAGGAAGTCTAATCTCAAACCTTTTGAGAGAGATATTTCAACTGAAATCTATCAGGCCGGTAACAAACCTTGTAATGGTGTTGCAGGTTTTAATTGTTACTTTCCTTTACGATCATATGGTTTCCGACCCACTTATGGTGTTGGTCACCAACCATACAGAGTAGTAGTACTTTCTTTTGAACTTCTA | | |
|  |  | **Genotype** | **Codon** | **Sanger sequencing diagram** |
|  |  | 452L | CTG | 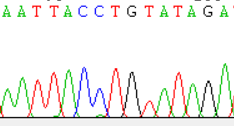 |
|  |  | 460N | AAT | 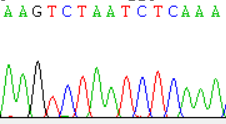 |
|  |  | 484A, 486F | GCA, TTT | 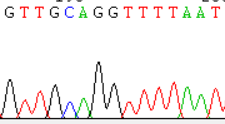 |
|  |  | 493R | CGA | 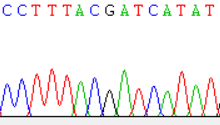 |
|  |  | 498R | CGA | 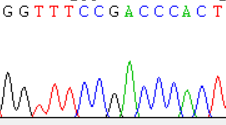 |
|  |  | 505H | CAC | 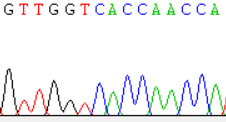 |
| 573 | BA.2 | GATGATTTTACAGGCTGCGTTATAGCTTGGAATTCTAACAAGCTTGATTCTAAGGTTGGTGGTAATTATAATTACCTGTATAGATTGTTTAGGAAGTCTAATCTCAAACCTTTTGAGAGAGATATTTCAACTGAAATCTATCAGGCCGGTAACAAACCTTGTAATGGTGTTGCAGGTTTTAATTGTTACTTTCCTTTACGATCATATGGTTTCCGACCCACTTATGGTGTTGGTCACCAACCATACAGAGTAGTAGTACTTTCTTTTGAACTTCTA | | |
|  |  | **Genotype** | **Codon** | **Sanger sequencing diagram** |
|  |  | 452L | CTG | 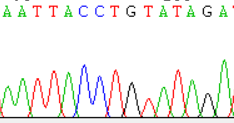 |
|  |  | 460N | AAT | 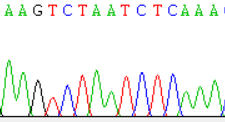 |
|  |  | 484A, 486F | GCA, TTT | 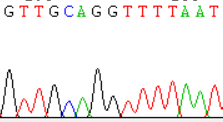 |
|  |  | 493R | CGA | 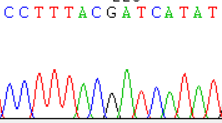 |
|  |  | 498R | CGA | 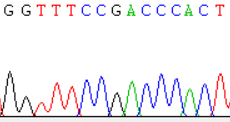 |
|  |  | 505H | CAC | 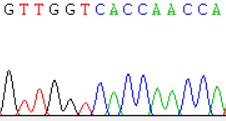 |
| 577 | BA.2 | GATGATTTTACAGGCTGCGTTATAGCTTGGAATTCTAACAAGCTTGATTCTAAGGTTGGTGGTAATTATAATTACCTGTATAGATTGTTTAGGAAGTCTAATCTCAAACCTTTTGAGAGAGATATTTCAACTGAAATCTATCAGGCCGGTAACAAACCTTGTAATGGTGTTGCAGGTTTTAATTGTTACTTTCCTTTACGATCATATGGTTTCCGACCCACTTATGGTGTTGGTCACCAACCATACAGAGTAGTAGTACTTTCTTTTGAACTTCTA | | |
|  |  | **Genotype** | **Codon** | **Sanger sequencing diagram** |
|  |  | 452L | CTG | 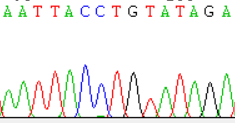 |
|  |  | 460N | AAT | 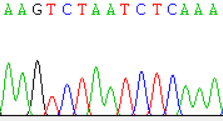 |
|  |  | 484A, 486F | GCA, TTT | 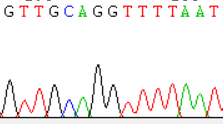 |
|  |  | 493R | CGA | 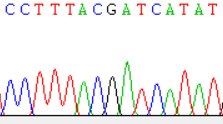 |
|  |  | 498R | CGA | 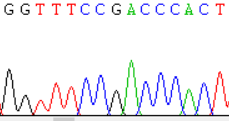 |
|  |  | 505H | CAC | 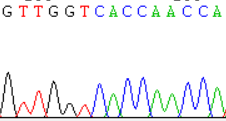 |
| 579 | BA.2 | GATGATTTTACAGGCTGCGTTATAGCTTGGAATTCTAACAAGCTTGATTCTAAGGTTGGTGGTAATTATAATTACCTGTATAGATTGTTTAGGAAGTCTAATCTCAAACCTTTTGAGAGAGATATTTCAACTGAAATCTATCAGGCCGGTAACAAACCTTGTAATGGTGTTGCAGGTTTTAATTGTTACTTTCCTTTACGATCATATGGTTTCCGACCCACTTATGGTGTTGGTCACCAACCATACAGAGTAGTAGTACTTTCTTTTGAACTTCTA | | |
|  |  | **Genotype** | **Codon** | **Sanger sequencing diagram** |
|  |  | 452L | CTG | 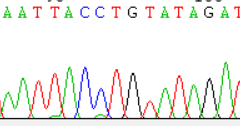 |
|  |  | 460N | AAT | 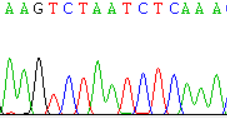 |
|  |  | 484A, 486F | GCA, TTT | 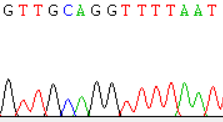 |
|  |  | 493R | CGA | 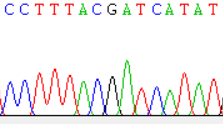 |
|  |  | 498R | CGA | 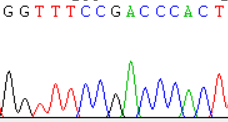 |
|  |  | 505H | CAC | 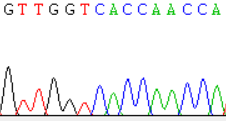 |
| 69 | BA.2 | GATGATTTTACAGGCTGCGTTATAGCTTGGAATTCTAACAAGCTTGATTCTAAGGTTGGTGGTAATTATAATTACCTGTATAGATTGTTTAGGAAGTCTAATCTCAAACCTTTTGAGAGAGATATTTCAACTGAAATCTATCAGGCCGGTAACAAACCTTGTAATGGTGTTGCAGGTTTTAATTGTTACTTTCCTTTACGATCATATGGTTTCCGACCCACTTATGGTGTTGGTCACCAACCATACAGAGTAGTAGTACTTTCTTTTGAACTTCTA | | |
|  |  | **Genotype** | **Codon** | **Sanger sequencing diagram** |
|  |  | 452L | CTG | 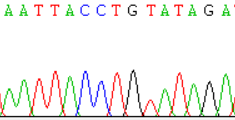 |
|  |  | 460N | AAT | 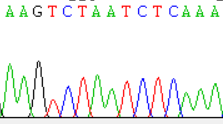 |
|  |  | 484A, 486F | GCA, TTT | 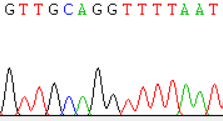 |
|  |  | 493R | CGA | 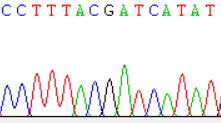 |
|  |  | 498R | CGA | 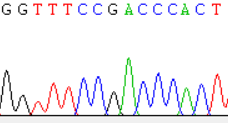 |
|  |  | 505H | CAC | 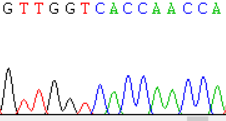 |
| 618 | BA.2 | GATGATTTTACAGGCTGCGTTATAGCTTGGAATTCTAACAAGCTTGATTCTAAGGTTGGTGGTAATTATAATTACCTGTATAGATTGTTTAGGAAGTCTAATCTCAAACCTTTTGAGAGAGATATTTCAACTGAAATCTATCAGGCCGGTAACAAACCTTGTAATGGTGTTGCAGGTTTTAATTGTTACTTTCCTTTACGATCATATGGTTTCCGACCCACTTATGGTGTTGGTCACCAACCATACAGAGTAGTAGTACTTTCTTTTGAACTTCTA | | |
|  |  | **Genotype** | **Codon** | **Sanger sequencing diagram** |
|  |  | 452L | CTG | 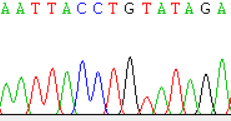 |
|  |  | 460N | AAT | 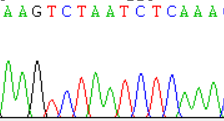 |
|  |  | 484A, 486F | GCA, TTT | 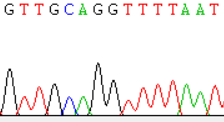 |
|  |  | 493R | CGA | 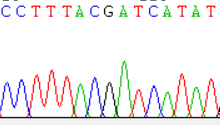 |
|  |  | 498R | CGA | 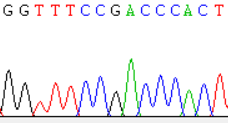 |
|  |  | 505H | CAC | 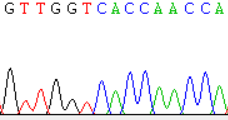 |
| 649 | BA.2 | GATGATTTTACAGGCTGCGTTATAGCTTGGAATTCTAACAAGCTTGATTCTAAGGTTGGTGGTAATTATAATTACCTGTATAGATTGTTTAGGAAGTCTAATCTCAAACCTTTTGAGAGAGATATTTCAACTGAAATCTATCAGGCCGGTAACAAACCTTGTAATGGTGTTGCAGGTTTTAATTGTTACTTTCCTTTACGATCATATGGTTTCCGACCCACTTATGGTGTTGGTCACCAACCATACAGAGTAGTAGTACTTTCTTTTGAACTTCTA | | |
|  |  | **Genotype** | **Codon** | **Sanger sequencing diagram** |
|  |  | 452L | CTG | 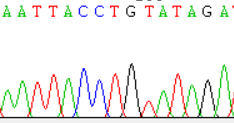 |
|  |  | 460N | AAT | 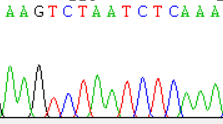 |
|  |  | 484A, 486F | GCA, TTT | 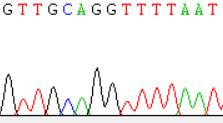 |
|  |  | 493R | CGA | 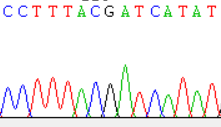 |
|  |  | 498R | CGA | 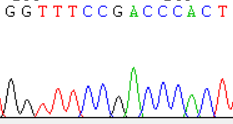 |
|  |  | 505H | CAC | 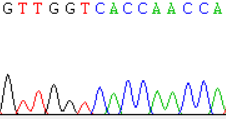 |
| M34 | BA.4/5 | GATGATTTTACAGGCTGCGTTATAGCTTGGAATTCTAACAAGCTTGATTCTAAGGTTGGTGGTAATTATAATTACCGGTATAGATTGTTTAGGAAGTCTAATCTCAAACCTTTTGAGAGAGATATTTCAACTGAAATCTATCAGGCCGGTAACAAACCTTGTAATGGTGTTGCAGGTGTTAATTGTTACTTTCCTTTACAATCATATGGTTTCCGACCCACTTATGGTGTTGGTCACCAACCATACAGAGTAGTAGTACTTTCTTTTGAACTTCTA | | |
|  |  | **Genotype** | **Codon** | **Sanger sequencing diagram** |
|  |  | 452R | CGG | 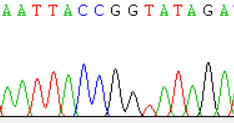 |
|  |  | 460N | AAT | 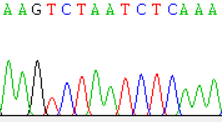 |
|  |  | 484A, 486V | GCA, GTT | 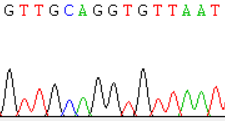 |
|  |  | 493Q | CAA | 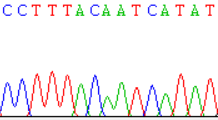 |
|  |  | 498R | CGA | 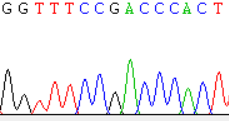 |
|  |  | 505H | CAC | 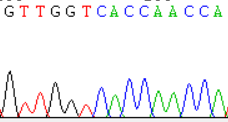 |
| 580 | BA.2 | GATGATTTTACAGGCTGCGTTATAGCTTGGAATTCTAACAAGCTTGATTCTAAGGTTGGTGGTAATTATAATTACCTGTATAGATTGTTTAGGAAGTCTAATCTCAAACCTTTTGAGAGAGATATTTCAACTGAAATCTATCAGGCCGGTAACAAACCTTGTAATGGTGTTGCAGGTTTTAATTGTTACTTTCCTTTACGATCATATGGTTTCCGACCCACTTATGGTGTTGGTCACCAACCATACAGAGTAGTAGTACTTTCTTTTGAACTTCTA | | |
|  |  | **Genotype** | **Codon** | **Sanger sequencing diagram** |
|  |  | 452L | CTG | 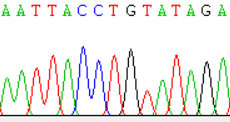 |
|  |  | 460N | AAT | 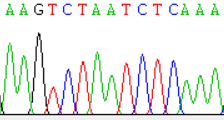 |
|  |  | 484A, 486F | GCA, TTT | 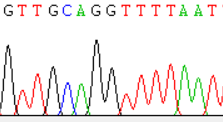 |
|  |  | 493R | CGA | 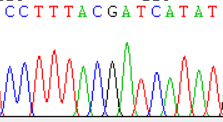 |
|  |  | 498R | CGA | 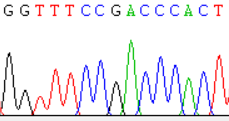 |
|  |  | 505H | CAC | 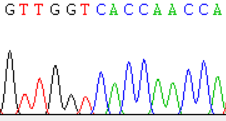 |
| 584 | BA.2 | GATGATTTTACAGGCTGCGTTATAGCTTGGAATTCTAACAAGCTTGATTCTAAGGTTGGTGGTAATTATAATTACCTGTATAGATTGTTTAGGAAGTCTAATCTCAAACCTTTTGAGAGAGATATTTCAACTGAAATCTATCAGGCCGGTAACAAACCTTGTAATGGTGTTGCAGGTTTTAATTGTTACTTTCCTTTACGATCATATGGTTTCCGACCCACTTATGGTGTTGGTCACCAACCATACAGAGTAGTAGTACTTTCTTTTGAACTTCTA | | |
|  |  | **Genotype** | **Codon** | **Sanger sequencing diagram** |
|  |  | 452L | CTG | 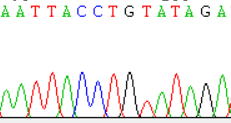 |
|  |  | 460N | AAT | 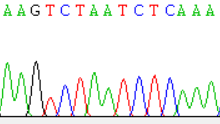 |
|  |  | 484A, 486F | GCA, TTT | 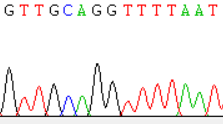 |
|  |  | 493R | CGA | 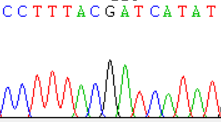 |
|  |  | 498R | CGA | 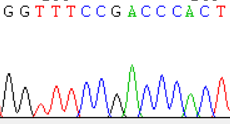 |
|  |  | 505H | CAC | 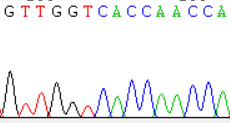 |
| 585 | BA.2 | GATGATTTTACAGGCTGCGTTATAGCTTGGAATTCTAACAAGCTTGATTCTAAGGTTGGTGGTAATTATAATTACCTGTATAGATTGTTTAGGAAGTCTAATCTCAAACCTTTTGAGAGAGATATTTCAACTGAAATCTATCAGGCCGGTAACAAACCTTGTAATGGTGTTGCAGGTTTTAATTGTTACTTTCCTTTACGATCATATGGTTTCCGACCCACTTATGGTGTTGGTCACCAACCATACAGAGTAGTAGTACTTTCTTTTGAACTTCTA | | |
|  |  | **Genotype** | **Codon** | **Sanger sequencing diagram** |
|  |  | 452L | CTG | 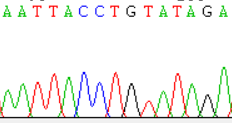 |
|  |  | 460N | AAT | 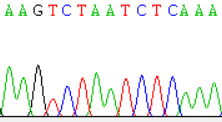 |
|  |  | 484A, 486F | GCA, TTT | 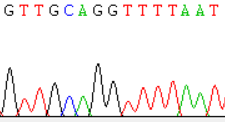 |
|  |  | 493R | CGA | 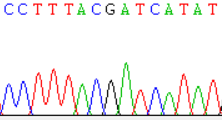 |
|  |  | 498R | CGA |  |
|  |  | 505H | CAC |  |
| 592 | BA.2 | GATGATTTTACAGGCTGCGTTATAGCTTGGAATTCTAACAAGCTTGATTCTAAGGTTGGTGGTAATTATAATTACCTGTATAGATTGTTTAGGAAGTCTAATCTCAAACCTTTTGAGAGAGATATTTCAACTGAAATCTATCAGGCCGGTAACAAACCTTGTAATGGTGTTGCAGGTTTTAATTGTTACTTTCCTTTACGATCATATGGTTTCCGACCCACTTATGGTGTTGGTCACCAACCATACAGAGTAGTAGTACTTTCTTTTGAACTTCTA | | |
|  |  | **Genotype** | **Codon** | **Sanger sequencing diagram** |
|  |  | 452L | CTG |  |
|  |  | 460N | AAT |  |
|  |  | 484A, 486F | GCA, TTT |  |
|  |  | 493R | CGA |  |
|  |  | 498R | CGA |  |
|  |  | 505H | CAC |  |
| 661 | BA.2 | GATGATTTTACAGGCTGCGTTATAGCTTGGAATTCTAACAAGCTTGATTCTAAGGTTGGTGGTAATTATAATTACCTGTATAGATTGTTTAGGAAGTCTAATCTCAAACCTTTTGAGAGAGATATTTCAACTGAAATCTATCAGGCCGGTAACAAACCTTGTAATGGTGTTGCAGGTTTTAATTGTTACTTTCCTTTACGATCATATGGTTTCCGACCCACTTATGGTGTTGGTCACCAACCATACAGAGTAGTAGTACTTTCTTTTGAACTTCTA | | |
|  |  | **Genotype** | **Codon** | **Sanger sequencing diagram** |
|  |  | 452L | CTG |  |
|  |  | 460N | AAT |  |
|  |  | 484A, 486F | GCA, TTT |  |
|  |  | 493R | CGA |  |
|  |  | 498R | CGA |  |
|  |  | 505H | CAC |  |
| 673 | BA.2 | GATGATTTTACAGGCTGCGTTATAGCTTGGAATTCTAACAAGCTTGATTCTAAGGTTGGTGGTAATTATAATTACCTGTATAGATTGTTTAGGAAGTCTAATCTCAAACCTTTTGAGAGAGATATTTCAACTGAAATCTATCAGGCCGGTAACAAACCTTGTAATGGTGTTGCAGGTTTTAATTGTTACTTTCCTTTACGATCATATGGTTTCCGACCCACTTATGGTGTTGGTCACCAACCATACAGAGTAGTAGTACTTTCTTTTGAACTTCTA | | |
|  |  | **Genotype** | **Codon** | **Sanger sequencing diagram** |
|  |  | 452L | CTG |  |
|  |  | 460N | AAT |  |
|  |  | 484A, 486F | GCA, TTT |  |
|  |  | 493R | CGA |  |
|  |  | 498R | CGA |  |
|  |  | 505H | CAC |  |
| 680 | BA.2 | GATGATTTTACAGGCTGCGTTATAGCTTGGAATTCTAACAAGCTTGATTCTAAGGTTGGTGGTAATTATAATTACCTGTATAGATTGTTTAGGAAGTCTAATCTCAAACCTTTTGAGAGAGATATTTCAACTGAAATCTATCAGGCCGGTAACAAACCTTGTAATGGTGTTGCAGGTTTTAATTGTTACTTTCCTTTACGATCATATGGTTTCCGACCCACTTATGGTGTTGGTCACCAACCATACAGAGTAGTAGTACTTTCTTTTGAACTTCTA | | |
|  |  | **Genotype** | **Codon** | **Sanger sequencing diagram** |
|  |  | 452L | CTG |  |
|  |  | 460N | AAT |  |
|  |  | 484A, 486F | GCA, TTT |  |
|  |  | 493R | CGA |  |
|  |  | 498R | CGA |  |
|  |  | 505H | CAC |  |
| 682 | BA.2 | GATGATTTTACAGGCTGCGTTATAGCTTGGAATTCTAACAAGCTTGATTCTAAGGTTGGTGGTAATTATAATTACCTGTATAGATTGTTTAGGAAGTCTAATCTCAAACCTTTTGAGAGAGATATTTCAACTGAAATCTATCAGGCCGGTAACAAACCTTGTAATGGTGTTGCAGGTTTTAATTGTTACTTTCCTTTACGATCATATGGTTTCCGACCCACTTATGGTGTTGGTCACCAACCATACAGAGTAGTAGTACTTTCTTTTGAACTTCTA | | |
|  |  | **Genotype** | **Codon** | **Sanger sequencing diagram** |
|  |  | 452L | CTG |  |
|  |  | 460N | AAT |  |
|  |  | 484A, 486F | GCA, TTT |  |
|  |  | 493R | CGA |  |
|  |  | 498R | CGA |  |
|  |  | 505H | CAC |  |
| 760 | BA.2 | GATGATTTTACAGGCTGCGTTATAGCTTGGAATTCTAACAAGCTTGATTCTAAGGTTGGTGGTAATTATAATTACCTGTATAGATTGTTTAGGAAGTCTAATCTCAAACCTTTTGAGAGAGATATTTCAACTGAAATCTATCAGGCCGGTAACAAACCTTGTAATGGTGTTGCAGGTTTTAATTGTTACTTTCCTTTACGATCATATGGTTTCCGACCCACTTATGGTGTTGGTCACCAACCATACAGAGTAGTAGTACTTTCTTTTGAACTTCTA | | |
|  |  | **Genotype** | **Codon** | **Sanger sequencing diagram** |
|  |  | 452L | CTG |  |
|  |  | 460N | AAT |  |
|  |  | 484A, 486F | GCA, TTT |  |
|  |  | 493R | CGA |  |
|  |  | 498R | CGA |  |
|  |  | 505H | CAC |  |
| 765 | BA.2 | GATGATTTTACAGGCTGCGTTATAGCTTGGAATTCTAACAAGCTTGATTCTAAGGTTGGTGGTAATTATAATTACCTGTATAGATTGTTTAGGAAGTCTAATCTCAAACCTTTTGAGAGAGATATTTCAACTGAAATCTATCAGGCCGGTAACAAACCTTGTAATGGTGTTGCAGGTTTTAATTGTTACTTTCCTTTACGATCATATGGTTTCCGACCCACTTATGGTGTTGGTCACCAACCATACAGAGTAGTAGTACTTTCTTTTGAACTTCTA | | |
|  |  | **Genotype** | **Codon** | **Sanger sequencing diagram** |
|  |  | 452L | CTG |  |
|  |  | 460N | AAT |  |
|  |  | 484A, 486F | GCA, TTT |  |
|  |  | 493R | CGA |  |
|  |  | 498R | CGA |  |
|  |  | 505H | CAC |  |
| 784 | BA.2 | GATGATTTTACAGGCTGCGTTATAGCTTGGAATTCTAACAAGCTTGATTCTAAGGTTGGTGGTAATTATAATTACCTGTATAGATTGTTTAGGAAGTCTAATCTCAAACCTTTTGAGAGAGATATTTCAACTGAAATCTATCAGGCCGGTAACAAACCTTGTAATGGTGTTGCAGGTTTTAATTGTTACTTTCCTTTACGATCATATGGTTTCCGACCCACTTATGGTGTTGGTCACCAACCATACAGAGTAGTAGTACTTTCTTTTGAACTTCTA | | |
|  |  | **Genotype** | **Codon** | **Sanger sequencing diagram** |
|  |  | 452L | CTG |  |
|  |  | 460N | AAT |  |
|  |  | 484A, 486F | GCA, TTT |  |
|  |  | 493R | CGA |  |
|  |  | 498R | CGA |  |
|  |  | 505H | CAC |  |
| 789 | BA.2 | GATGATTTTACAGGCTGCGTTATAGCTTGGAATTCTAACAAGCTTGATTCTAAGGTTGGTGGTAATTATAATTACCTGTATAGATTGTTTAGGAAGTCTAATCTCAAACCTTTTGAGAGAGATATTTCAACTGAAATCTATCAGGCCGGTAACAAACCTTGTAATGGTGTTGCAGGTTTTAATTGTTACTTTCCTTTACGATCATATGGTTTCCGACCCACTTATGGTGTTGGTCACCAACCATACAGAGTAGTAGTACTTTCTTTTGAACTTCTA | | |
|  |  | **Genotype** | **Codon** | **Sanger sequencing diagram** |
|  |  | 452L | CTG |  |
|  |  | 460N | AAT |  |
|  |  | 484A, 486F | GCA, TTT |  |
|  |  | 493R | CGA |  |
|  |  | 498R | CGA |  |
|  |  | 505H | CAC |  |
| 816 | BA.2 | GATGATTTTACAGGCTGCGTTATAGCTTGGAATTCTAACAAGCTTGATTCTAAGGTTGGTGGTAATTATAATTACCTGTATAGATTGTTTAGGAAGTCTAATCTCAAACCTTTTGAGAGAGATATTTCAACTGAAATCTATCAGGCCGGTAACAAACCTTGTAATGGTGTTGCAGGTTTTAATTGTTACTTTCCTTTACGATCATATGGTTTCCGACCCACTTATGGTGTTGGTCACCAACCATACAGAGTAGTAGTACTTTCTTTTGAACTTCTA | | |
|  |  | **Genotype** | **Codon** | **Sanger sequencing diagram** |
|  |  | 452L | CTG |  |
|  |  | 460N | AAT |  |
|  |  | 484A, 486F | GCA, TTT |  |
|  |  | 493R | CGA |  |
|  |  | 498R | CGA |  |
|  |  | 505H | CAC |  |
| 860 | BA.2 | GATGATTTTACAGGCTGCGTTATAGCTTGGAATTCTAACAAGCTTGATTCTAAGGTTGGTGGTAATTATAATTACCTGTATAGATTGTTTAGGAAGTCTAATCTCAAACCTTTTGAGAGAGATATTTCAACTGAAATCTATCAGGCCGGTAACAAACCTTGTAATGGTGTTGCAGGTTTTAATTGTTACTTTCCTTTACGATCATATGGTTTCCGACCCACTTATGGTGTTGGTCACCAACCATACAGAGTAGTAGTACTTTCTTTTGAACTTCTA | | |
|  |  | **Genotype** | **Codon** | **Sanger sequencing diagram** |
|  |  | 452L | CTG |  |
|  |  | 460N | AAT |  |
|  |  | 484A, 486F | GCA, TTT |  |
|  |  | 493R | CGA |  |
|  |  | 498R | CGA |  |
|  |  | 505H | CAC |  |
| 873 | BA.2 | GATGATTTTACAGGCTGCGTTATAGCTTGGAATTCTAACAAGCTTGATTCTAAGGTTGGTGGTAATTATAATTACCTGTATAGATTGTTTAGGAAGTCTAATCTCAAACCTTTTGAGAGAGATATTTCAACTGAAATCTATCAGGCCGGTAACAAACCTTGTAATGGTGTTGCAGGTTTTAATTGTTACTTTCCTTTACGATCATATGGTTTCCGACCCACTTATGGTGTTGGTCACCAACCATACAGAGTAGTAGTACTTTCTTTTGAACTTCTA | | |
|  |  | **Genotype** | **Codon** | **Sanger sequencing diagram** |
|  |  | 452L | CTG |  |
|  |  | 460N | AAT |  |
|  |  | 484A, 486F | GCA, TTT |  |
|  |  | 493R | CGA |  |
|  |  | 498R | CGA |  |
|  |  | 505H | CAC |  |
| 877 | BA.2 | GATGATTTTACAGGCTGCGTTATAGCTTGGAATTCTAACAAGCTTGATTCTAAGGTTGGTGGTAATTATAATTACCTGTATAGATTGTTTAGGAAGTCTAATCTCAAACCTTTTGAGAGAGATATTTCAACTGAAATCTATCAGGCCGGTAACAAACCTTGTAATGGTGTTGCAGGTTTTAATTGTTACTTTCCTTTACGATCATATGGTTTCCGACCCACTTATGGTGTTGGTCACCAACCATACAGAGTAGTAGTACTTTCTTTTGAACTTCTA | | |
|  |  | **Genotype** | **Codon** | **Sanger sequencing diagram** |
|  |  | 452L | CTG |  |
|  |  | 460N | AAT |  |
|  |  | 484A, 486F | GCA, TTT |  |
|  |  | 493R | CGA |  |
|  |  | 498R | CGA |  |
|  |  | 505H | CAC |  |
| 884 | BA.2 | GATGATTTTACAGGCTGCGTTATAGCTTGGAATTCTAACAAGCTTGATTCTAAGGTTGGTGGTAATTATAATTACCTGTATAGATTGTTTAGGAAGTCTAATCTCAAACCTTTTGAGAGAGATATTTCAACTGAAATCTATCAGGCCGGTAACAAACCTTGTAATGGTGTTGCAGGTTTTAATTGTTACTTTCCTTTACGATCATATGGTTTCCGACCCACTTATGGTGTTGGTCACCAACCATACAGAGTAGTAGTACTTTCTTTTGAACTTCTA | | |
|  |  | **Genotype** | **Codon** | **Sanger sequencing diagram** |
|  |  | 452L | CTG |  |
|  |  | 460N | AAT |  |
|  |  | 484A, 486F | GCA, TTT |  |
|  |  | 493R | CGA |  |
|  |  | 498R | CGA |  |
|  |  | 505H | CAC |  |
| 885 | BA.2 | GATGATTTTACAGGCTGCGTTATAGCTTGGAATTCTAACAAGCTTGATTCTAAGGTTGGTGGTAATTATAATTACCTGTATAGATTGTTTAGGAAGTCTAATCTCAAACCTTTTGAGAGAGATATTTCAACTGAAATCTATCAGGCCGGTAACAAACCTTGTAATGGTGTTGCAGGTTTTAATTGTTACTTTCCTTTACGATCATATGGTTTCCGACCCACTTATGGTGTTGGTCACCAACCATACAGAGTAGTAGTACTTTCTTTTGAACTTCTA | | |
|  |  | **Genotype** | **Codon** | **Sanger sequencing diagram** |
|  |  | 452L | CTG |  |
|  |  | 460N | AAT |  |
|  |  | 484A, 486F | GCA, TTT |  |
|  |  | 493R | CGA |  |
|  |  | 498R | CGA |  |
|  |  | 505H | CAC |  |
| 93 | BA.2 | GATGATTTTACAGGCTGCGTTATAGCTTGGAATTCTAACAAGCTTGATTCTAAGGTTGGTGGTAATTATAATTACCTGTATAGATTGTTTAGGAAGTCTAATCTCAAACCTTTTGAGAGAGATATTTCAACTGAAATCTATCAGGCCGGTAACAAACCTTGTAATGGTGTTGCAGGTTTTAATTGTTACTTTCCTTTACGATCATATGGTTTCCGACCCACTTATGGTGTTGGTCACCAACCATACAGAGTAGTAGTACTTTCTTTTGAACTTCTA | | |
|  |  | **Genotype** | **Codon** | **Sanger sequencing diagram** |
|  |  | 452L | CTG |  |
|  |  | 460N | AAT |  |
|  |  | 484A, 486F | GCA, TTT |  |
|  |  | 493R | CGA |  |
|  |  | 498R | CGA |  |
|  |  | 505H | CAC |  |
| 912 | BA.2 | GATGATTTTACAGGCTGCGTTATAGCTTGGAATTCTAACAAGCTTGATTCTAAGGTTGGTGGTAATTATAATTACCTGTATAGATTGTTTAGGAAGTCTAATCTCAAACCTTTTGAGAGAGATATTTCAACTGAAATCTATCAGGCCGGTAACAAACCTTGTAATGGTGTTGCAGGTTTTAATTGTTACTTTCCTTTACGATCATATGGTTTCCGACCCACTTATGGTGTTGGTCACCAACCATACAGAGTAGTAGTACTTTCTTTTGAACTTCTA | | |
|  |  | **Genotype** | **Codon** | **Sanger sequencing diagram** |
|  |  | 452L | CTG |  |
|  |  | 460N | AAT |  |
|  |  | 484A, 486F | GCA, TTT |  |
|  |  | 493R | CGA |  |
|  |  | 498R | CGA |  |
|  |  | 505H | CAC |  |
| 913 | BA.2 | GATGATTTTACAGGCTGCGTTATAGCTTGGAATTCTAACAAGCTTGATTCTAAGGTTGGTGGTAATTATAATTACCTGTATAGATTGTTTAGGAAGTCTAATCTCAAACCTTTTGAGAGAGATATTTCAACTGAAATCTATCAGGCCGGTAACAAACCTTGTAATGGTGTTGCAGGTTTTAATTGTTACTTTCCTTTACGATCATATGGTTTCCGACCCACTTATGGTGTTGGTCACCAACCATACAGAGTAGTAGTACTTTCTTTTGAACTTCTA | | |
|  |  | **Genotype** | **Codon** | **Sanger sequencing diagram** |
|  |  | 452L | CTG |  |
|  |  | 460N | AAT |  |
|  |  | 484A, 486F | GCA, TTT |  |
|  |  | 493R | CGA |  |
|  |  | 498R | CGA |  |
|  |  | 505H | CAC |  |
| 949 | BA.2 | GATGATTTTACAGGCTGCGTTATAGCTTGGAATTCTAACAAGCTTGATTCTAAGGTTGGTGGTAATTATAATTACCTGTATAGATTGTTTAGGAAGTCTAATCTCAAACCTTTTGAGAGAGATATTTCAACTGAAATCTATCAGGCCGGTAACAAACCTTGTAATGGTGTTGCAGGTTTTAATTGTTACTTTCCTTTACGATCATATGGTTTCCGACCCACTTATGGTGTTGGTCACCAACCATACAGAGTAGTAGTACTTTCTTTTGAACTTCTA | | |
|  |  | **Genotype** | **Codon** | **Sanger sequencing diagram** |
|  |  | 452L | CTG |  |
|  |  | 460N | AAT |  |
|  |  | 484A, 486F | GCA, TTT |  |
|  |  | 493R | CGA |  |
|  |  | 498R | CGA |  |
|  |  | 505H | CAC |  |
| 961 | BA.2 | GATGATTTTACAGGCTGCGTTATAGCTTGGAATTCTAACAAGCTTGATTCTAAGGTTGGTGGTAATTATAATTACCTGTATAGATTGTTTAGGAAGTCTAATCTCAAACCTTTTGAGAGAGATATTTCAACTGAAATCTATCAGGCCGGTAACAAACCTTGTAATGGTGTTGCAGGTTTTAATTGTTACTTTCCTTTACGATCATATGGTTTCCGACCCACTTATGGTGTTGGTCACCAACCATACAGAGTAGTAGTACTTTCTTTTGAACTTCTA | | |
|  |  | **Genotype** | **Codon** | **Sanger sequencing diagram** |
|  |  | 452L | CTG |  |
|  |  | 460N | AAT |  |
|  |  | 484A, 486F | GCA, TTT |  |
|  |  | 493R | CGA |  |
|  |  | 498R | CGA |  |
|  |  | 505H | CAC |  |
| 984 | BA.2 | GATGATTTTACAGGCTGCGTTATAGCTTGGAATTCTAACAAGCTTGATTCTAAGGTTGGTGGTAATTATAATTACCTGTATAGATTGTTTAGGAAGTCTAATCTCAAACCTTTTGAGAGAGATATTTCAACTGAAATCTATCAGGCCGGTAACAAACCTTGTAATGGTGTTGCAGGTTTTAATTGTTACTTTCCTTTACGATCATATGGTTTCCGACCCACTTATGGTGTTGGTCACCAACCATACAGAGTAGTAGTACTTTCTTTTGAACTTCTA | | |
|  |  | **Genotype** | **Codon** | **Sanger sequencing diagram** |
|  |  | 452L | CTG |  |
|  |  | 460N | AAT |  |
|  |  | 484A, 486F | GCA, TTT |  |
|  |  | 493R | CGA |  |
|  |  | 498R | CGA |  |
|  |  | 505H | CAC |  |
| M90 | BA.4/5 | GATGATTTTACAGGCTGCGTTATAGCTTGGAATTCTAACAAGCTTGATTCTAAGGTTGGTGGTAATTATAATTACCGGTATAGATTGTTTAGGAAGTCTAATCTCAAACCTTTTGAGAGAGATATTTCAACTGAAATCTATCAGGCCGGTAACAAACCTTGTAATGGTGTTGCAGGTGTTAATTGTTACTTTCCTTTACAATCATATGGTTTCCGACCCACTTATGGTGTTGGTCACCAACCATACAGAGTAGTAGTACTTTCTTTTGAACTTCTA | | |
|  |  | **Genotype** | **Codon** | **Sanger sequencing diagram** |
|  |  | 452R | CGG |  |
|  |  | 460N | AAT |  |
|  |  | 484A, 486V | GCA, GTT |  |
|  |  | 493Q | CAA |  |
|  |  | 498R | CGA |  |
|  |  | 505H | CAC |  |
| M84 | BA.4/5 | GATGATTTTACAGGCTGCGTTATAGCTTGGAATTCTAACAAGCTTGATTCTAAGGTTGGTGGTAATTATAATTACCGGTATAGATTGTTTAGGAAGTCTAATCTCAAACCTTTTGAGAGAGATATTTCAACTGAAATCTATCAGGCCGGTAACAAACCTTGTAATGGTGTTGCAGGTGTTAATTGTTACTTTCCTTTACAATCATATGGTTTCCGACCCACTTATGGTGTTGGTCACCAACCATACAGAGTAGTAGTACTTTCTTTTGAACTTCTA | | |
|  |  | **Genotype** | **Codon** | **Sanger sequencing diagram** |
|  |  | 452R | CGG |  |
|  |  | 460N | AAT |  |
|  |  | 484A, 486V | GCA, GTT |  |
|  |  | 493Q | CAA |  |
|  |  | 498R | CGA |  |
|  |  | 505H | CAC |  |
